# Error-based Extraction of States and Energy Landscapes from Experimental Single-Molecule Time-Series

**DOI:** 10.1038/srep09174

**Published:** 2015-03-17

**Authors:** J. Nicholas Taylor, Chun-Biu Li, David R. Cooper, Christy F. Landes, Tamiki Komatsuzaki

**Affiliations:** 1 Research Institute for Electronic Science, Hokkaido University, Kita 20 Nishi 10, Kita-Ku, Sapporo 001-0020 Japan; 2 Department of Chemistry, Rice University, P.O. Box 1892, Houston. TX 77005

## Abstract

Characterization of states, the essential components of the underlying energy landscapes, is one of the most intriguing subjects in single-molecule (SM) experiments due to the existence of noise inherent to the measurements. Here we present a method to extract the underlying state sequences from experimental SM time-series. Taking into account empirical error and the finite sampling of the time-series, the method extracts a steady-state network which provides an approximation of the underlying effective free energy landscape. The core of the method is the application of rate-distortion theory from information theory, allowing the individual data points to be assigned to multiple states simultaneously. We demonstrate the method's proficiency in its application to simulated trajectories as well as to experimental SM fluorescence resonance energy transfer (FRET) trajectories obtained from isolated agonist binding domains of the AMPA receptor, an ionotropic glutamate receptor that is prevalent in the central nervous system.

Single-molecule (SM) measurements are standard experimental techniques in many fields of study^1^,^2^,^3^,^4^,^5^,^6^,^7^,^8^, and provide valuable information not only on the distribution of the observable but also on the dynamical pathways molecules may take in route to comprising the distribution. SM time-series measurements enable us to identify the sequences of states and construct the state-to-state network^9^,^10^,^11^,^12^,^13^, from which the underlying effective free energy landscape the single molecules experience can be inferred^14^,^15^. As such, identification of states along noisy time-series is one of the most prevalent subjects in SM time-series measurements. Among state identification methods, perhaps the most widely used for SM trajectories is the hidden-Markov model (HMM)^16^,^17^. The HMM procedure involves making an initial guess as to the number of states underlying the system. The stationary and dynamical properties of the underlying states are then extracted via a parameter optimization procedure, yielding the maximum likelihood HMM. The variational Bayes approach^18^ is a more sophisticated HMM that avoids some of the assumptions made in previous HMM applications – namely the need for the assumption of the number of states underlying the data. Local equilibrium state analysis^12^,^13^ is also aimed at extracting the sequence of states, each of which is locally equilibrated, along a time-series, resulting in their Markovian network, and at inferring an effective free energy landscape. Still other methods seek to move past Markovian assumptions, extracting non-Markovian memory kernels directly from the data^19^.

Free energy landscapes are usually computed by projecting the landscape onto some chosen coordinates. However, such projection has been known to mask the real complexity of the underlying conformational networks and sometimes yield misleading results^20^. Disconnectivity graphs^21^,^22^,^23^ have been developed to visualize and capture the hierarchical organization of minima and saddles on energy landscapes free from any projections, especially for relatively small systems. However, the original procedure for disconnectivity graphs may not adequately manage the existence of multiple pathways connecting some pairs of states in a complex conformational network. Transition disconnectivity graphs^24^,^25^ (TRDGs) based on the max-flow min-cut theorem^26^,^27^ from network theory seek to resolve this issue, and are a promising protocol in inferring effective energy landscapes derived from state-to-state Markovian networks at equilibrium. TRDG methods were designed for application to conformational space networks derived from noise-free computer simulations, and have yet to be applied to noisy experimental data that are subject to experimental errors and finite sampling.

Care should be taken in the quantification of SM experiments, as they often suffer from low signal-to-noise ratio. A recent change-point identification method revealed that the possibility of misidentifying the underlying SM kinetics exists when a simpler thresholding procedure is used to determine the states or levels comprising the time-series^28^. This was due to large, fast fluctuation of the noise contaminating the signals. It is therefore of crucial importance in SM measurements to develop methods that avoid making such misidentifications and to capture the relevant information concerning the SM kinetics from noisy signals. A variety of single-molecule analysis and denoising methods exist. Photon-by-photon methods use information theory to detect intensity change points in SM trajectories^29^ and to bin the data to constant error precision^30^, allowing for the subsequent deconvolution of the empirical error from the observable distribution. Using these methods, kinetic rates were determined without the need for state identification^31^. Wavelet denoising methods^32^,^33^ have been used to remove a portion of the empirical errors from uniformly binned SM photon signals. For noisy SM time-series of finite lengths, one must consider not only the contributions of empirical errors arising from various sources, but also the errors introduced by the finiteness of the time-series returned by the measurements.

The complications of errors in reconstructing a network, from which one can infer an effective free energy landscape using the TRDG protocol^24^,^25^, in terms of noisy SM FRET experiments are illustrated in Fig. 1. The segmentation procedure of local equilibrium state analysis^12^ is used to construct the state-to-state network directly along the time-series. In this demonstration we use a SM FRET efficiency trajectory acquired from a nitrowillardiine-bound agonist binding domain (ABD) of an AMPA receptor (GluA2)^34^, but we note that the procedure does not rely on the nature of the physical quantity being observed. Other physical quantities, such as fluorescence intensity or donor-acceptor distance are also appropriate as long as the error in the observable is appropriately considered. In a SM FRET experiment, photons are detected from both the donor (green ellipse) and acceptor (red ellipse) fluororphores attached to the molecule of interest – Fig. 1A depicts the nitrowillardiine-bound ABD of the GluA2 AMPA receptor^35^. Arrival times of each emitted photon are recorded (Fig. 1B), and the photon counts are binned, e.g., in uniform time intervals or in constant error intervals^30^. Binned photons are used to calculate a physical quantity (FRET efficiency in this demonstration) vs. time (Fig. 1C). The first step in the extraction of the underlying states along the time course is the segmentation of the time-series. Local probability mass functions (pmfs) are then constructed from the short segments of uniform length. The pmfs of the segments highlighted in blue and red in Fig. 1C are shown in Fig. 1D. Because the projection of motion in multiple dimensions onto a one-dimensional quantity such as FRET efficiency may result in a degeneracy problem, i.e., multiple underlying states yielding the same or similar efficiencies, local pmfs are used to lift any degeneracy as much as possible. (See Section S8 in the Supplementary Information (SI) for more detail.) Once the segment pmfs are obtained, similarity measures are calculated among all segment pmfs for use in clustering the segments into a steady-state network. The measure used is the Kantorovich distance^36^, illustrated by the shaded area between the cumulative distribution functions (cdfs) corresponding to the blue and red segments shown in Fig. 1E. The Kantorovich metric^34^ is used because it does not require the use of binning in computing the distance, and is therefore free from any artifact in choosing the bin size^12^, which is crucial, especially in the case of finitely sampled data. Furthermore, previous studies^12^ showed the Kantorovich distance to capture the actual distance between conformations in computer simulated data better than the Hellinger distance or the relative entropy. An underlying assumption in clustering the segments is that those arising from the same underlying state have small distances and segments arising from different underlying states have larger distances.

Clustering segments obtained from empirical data is hindered by empirical and finite sampling errors. In order to visualize the difficulties that arise from sources of error, the set of pairwise Kantorovich distances among the segments in the empirical trajectory (Fig. 1C) were mapped to principal coordinates with classical multidimensional scaling^37^. Such an algorithm places the objects (i.e., segments) in a multidimensional space such that the distance relationships among the objects are preserved as well as possible in a lower dimension. Error bars were generated as follows: from each segment 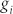 containing the set of *N* data points ***x****_i_* = {*x*_1_, …, *x_N_*}, *N* resamples were taken from the set ***x****_i_* with replacement, generating the bootstrapped segment 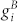 containing the set of points 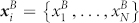. Each of the bootstrapped points 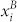 was then sampled from its empirical error distribution (e.g., a normal distribution centered at 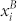), producing the set of points 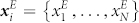. A pmf 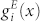 was then constructed from the set 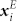, yielding a possible realization of the segment pmf, 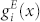 that contains contributions from sampling and empirical errors. Pairwise Kantorovich distances were calculated among all the segment pmfs 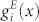, from which a new set of principal coordinate values were calculated. Repeating this process 100 times yields set of possible values for each segment in the principal coordinate space, from which the confidence intervals shown in Fig. 1F were estimated. Examination of Fig. 1F suggests that three states exist (colored circles), but assignment of many segments to a particular state is hampered by large errors and state overlap. Therefore, in order to obtain a steady-state network that yields an appropriate effective energy landscape, as shown in the TRDG in Fig. 1E, a clustering method that is amenable to the incorporation of errors is necessary for any application to noisy SM signals.

Clustering is a procedure in which a system is reduced from a large number of data points to a smaller number of clusters that embody regularities within the data set. In this light, we may view clustering as a form of *compression*. Furthermore, grouping elements that are similar but not identical into the same cluster results in *distortion*. Increasing the number of clusters describing the data set subsequently decreases distortion, but a small decrease in distortion may not be worth the ‘price’ paid in decreased compression. Clustering a data set may then be viewed as a tradeoff between compression and distortion. Rate-distortion theory is an information-theoretical technique developed by Shannon for use in communications^38^,^39^. Specifically, the theory provides a mathematical framework to determine the maximum achievable level of compression for a data set at a desired level of distortion. Rate-distortion theory then addresses the main objective of clustering, and the original formulation has since been enhanced towards this end, leading to alternative formulations such as the information bottleneck method^40^ and multi-information-based clustering^41^.

The power of clustering with rate-distortion theory lies in its use of soft clustering. In contrast to standard (i.e., hard) clustering, soft clustering allows data points to exist in multiple clusters, e.g., a cluster (i.e., state) *S_k_* is assigned to segment 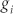 with conditional probability 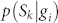. Soft clustering thus reflects the existence of uncertainty in state assignments arising from experimental errors, small numbers of data points, rare transitions from one state to another, *etc*. These conditional probabilities may be used to generate not only the most probable state sequence *S*_1_*S*_2_*S*_2_… (i.e., choosing *S_k_* at each segment 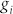 with 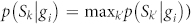, but many realizations of state sequences by randomly sampling from the conditional probabilities of each state at each segment. The various properties that are calculated from the most probable sequence may be calculated from every realization, yielding the most probable value of the properties as well as an estimation of their errors. For example, a state's escape time is commonly estimated by compiling residence times in the state and finding their mean. Performing this operation on many state sequence realizations yields a distribution of escape times from which the error can be inferred.

Here we develop a method combining the segmentation procedure of local equilibrium state analysis^12^ with rate-distortion theory to construct a steady-state network along a noisy two-color SM FRET experiment. Our method avoids several assumptions, namely the need to assume the number of states underlying the system as well as the need to assume properties of the states' distributions along the observable coordinate. In the construction of the network, our method must cope with experimental noise and the finiteness of the sampled data points. We describe a bootstrapping method designed to capture and quantify the effects of these errors. Through the application of the TRDG protocol^24^,^25^ to the steady-state network, we extract an energy landscape that accounts for the existence of multiple transition pathways. We term this new method Segmentation and Clustering with Information for Single-Molecule time-series (SCISM). After briefly describing rate-distortion theory, we concisely discuss the results of two simulated systems. An overdamped Langevin diffusion simulation was used to assess the performance of the SCISM procedure vs. several Gaussian/non-Gaussian noise levels, and a photon-by-photon simulation was used to validate the procedure under statistical conditions mimicking those of a SM FRET experiment. We then apply our method to experimental SM FRET data obtained from isolated agonist binding domains (ABDs) of the AMPA receptor^34^,^42^ while bound to a full agonist, a partial agonist, and an antagonist, extracting steady-state networks and effective energy landscapes for each of the systems. We discuss the relationship between the topographies of the energy landscapes and the ion channel activity, revealing new information about the activation mechanism of the ion channel.

## Results

We use rate-distortion theory to cluster, or compress, the set of *N* segments 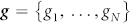 into a smaller set of *N_s_* states 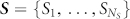 *via* the minimization of a functional expressing the tradeoff between compression and distortion.
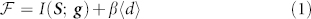
Here, the compression *I*(***S****;****g***) is the mutual information between the set of states ***S*** and the set of segment pmfs ***g***. The distortion 〈*d*〉, is the mean distance among all segment pmfs within each state and averaged over all states, and is constrained by the Lagrange multiplier *β*. The functional is minimized *via* an iterative calculation, returning results for a particular *β* and *N_s_*. The procedure requires initialization at several different values of *β* and *N_s_*, thereby returning a set of possible models. In selecting the appropriate model, we select the simplest model that best fits the data without overfitting it. This is achieved by determining the amount of distortion arising from errors, and thus determining the maximum *N_s_* the data will allow. See Methods and the SI for complete procedural details.

The proficiency of the SCISM procedure was assessed using two simulated systems. The first system, overdamped Langevin diffusion along a potential of mean force containing 5 minima (i.e., states), was used to examine the performance of the SCISM procedure vs. signal-to-noise ratio. Specifically, we tested the method against a broad range of signal-to-noise ratios and found SCISM to accurately return state distributions, escape times, and the TRDGs for experimentally reasonable signal-to-noise ratios. We also found that, as the signal-to-noise ratio decreases, the SCISM procedure begins to return fewer states than are present in the model system, finally returning a single state when the signal-to-noise ratio is large enough to obscure the underlying system. The results for this system not only establish guidelines for the use of the SCISM procedure in terms of the experimental signal-to-noise ratio, but also demonstrate that overfitting the system is prohibited by the model selection procedure. See SI Sections S1 and S2 for complete details concerning the simulation and the results.

The second simulated system is one mimicking the statistical conditions of a SM FRET experiment. SM FRET trajectories were constructed photon-by-photon such that the simulated system contains errors arising from photon counting, background contamination, and donor-to-acceptor crosstalk. The properties of the system, including the states' distributions and escape times, were extracted with precision, as all true properties were within the errors of the extracted properties. See SI Section 3 for a complete discussion of the simulation and results.

### Single-Molecule FRET measurements of AMPA ABDs

**Figure 1:**
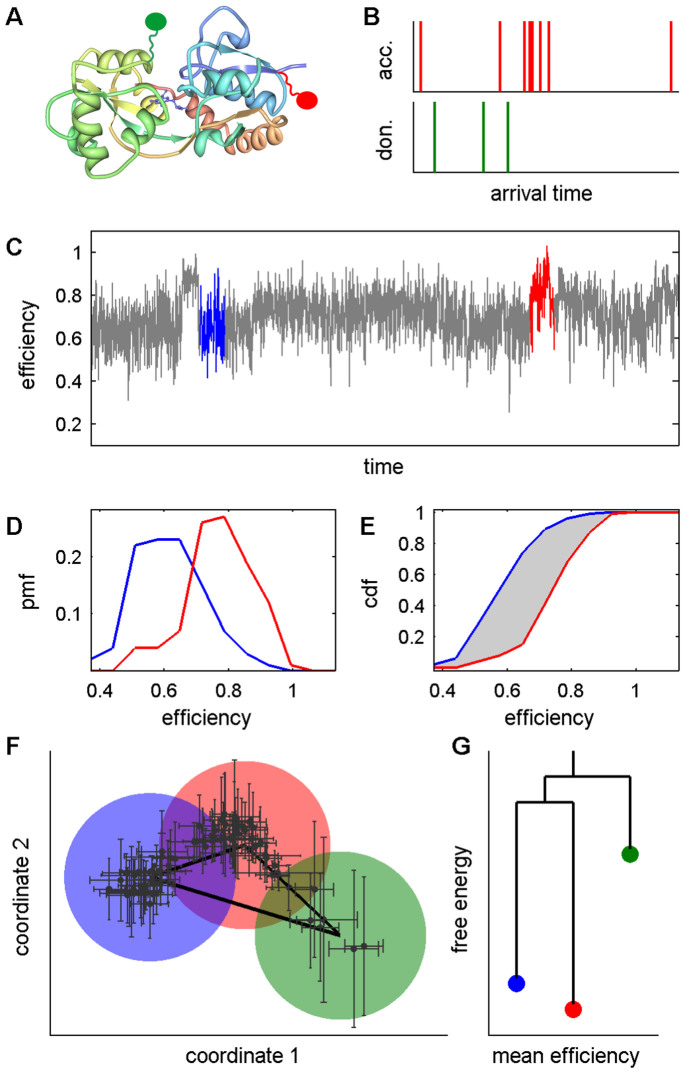
The complications of error in constructing TRDGs from experimental time-series. (A) Nitrowillardiine-bound GluA2 receptor ABD^33^ labeled with donor (green) and acceptor (red) flurorphores. (B) Arrival times of detected photons. (C) Uniformly binned FRET efficiency trajectory from the experimental SM FRET results for the nitrowillardiine-bound ABDs^32^. (D) The pmfs of the segments highlighted in blue and red in (C). (E) The area of the shaded region between the cdfs, corresponding to the pmfs in (D), is the Kantorovich distance between this pair of segments. (F) A 2-dimensional mapping of the distances among all the segments in the trajectory. Error bars include the contributions of empirical and sampling errors. The mapping suggests an underlying network comprised of 3 states (colored circles, heavy black lines), which is used to construct the effective energy landscape illustrated by the TRDG shown in (G). See Text and SI for full details.

AMPA receptors are tetrameric, ionotropic glutamate receptors comprised of extracellular N-terminal and ABDs, transmembrane domains, and intracellular C-terminal domains. Binding agonists such as glutamate triggers conformational shifts in the ABDs, leading to the activation of the ion channel. The allosteric mechanism by which channel activation occurs has been a subject of much recent interest considering that these receptors are the most abundant in the central nervous system and are implicated in a host of neurodegenerative disorders. The first step in channel activation is agonist binding; as such the ABD has been central to many investigations. As shown by the crystal structure in Fig. 1A, the ABD is bi-lobed forming a cleft with a central binding site. X-ray structures^43^ of the apo form of the ABD display an open cleft while those of agonist-bound forms have a shorter cleft distance, suggesting that cleft closure controls channel activation. Exceptions however, such as agonists that only partially activate the ion channel but whose x-ray structures show a small cleft distance^44^, indicate that this interpretation provides an incomplete picture of the activation mechanism.

X-ray structures provide only a static image of the ABD; a dynamical perspective is needed if the activation mechanism is to be well understood. Molecular dynamics simulations^45^,^46^ of the apo form displayed a generally open cleft as well as energetically inexpensive access multiple free energy minima along a 2-dimensional intra-cleft distance coordinate. Simulations of the glutamate-bound form exhibited a smaller cleft distance and a narrow free energy minimum corresponding to a closed-cleft conformation, but also displayed shallower free energy minima at larger cleft distances. This suggests a more dynamic picture in which channel activation is governed not simply by the degree of cleft closure, but by the agonist's ability to ‘hold’ the cleft closed through strong interactions with the lobes of the ABD. SM FRET experiments^42^ offered empirical evidence for this theoretical prediction, as multiple conformational states were observed in isolated apo and glutamate-bound ABDs. Additional SM FRET experiments^34^ for a group of partial agonists and antagonists provided further evidence to support this dynamical perspective of channel activation. Although dynamical aspects of the apo and glutamate-bound forms were discussed in terms of state-to-state kinetics and autocorrelation decay times^42^, these interpretations do not offer information concerning the energy landscapes associated with the ABD. Furthermore, few of these aspects were explored for the results of the partial agonists and antagonists presented in Ref. 34. We demonstrate that our new method provides a new and comprehensive interpretation on the basis of energy landscapes for these experimental results, deepening our understanding of the system.

**Figure 2:**
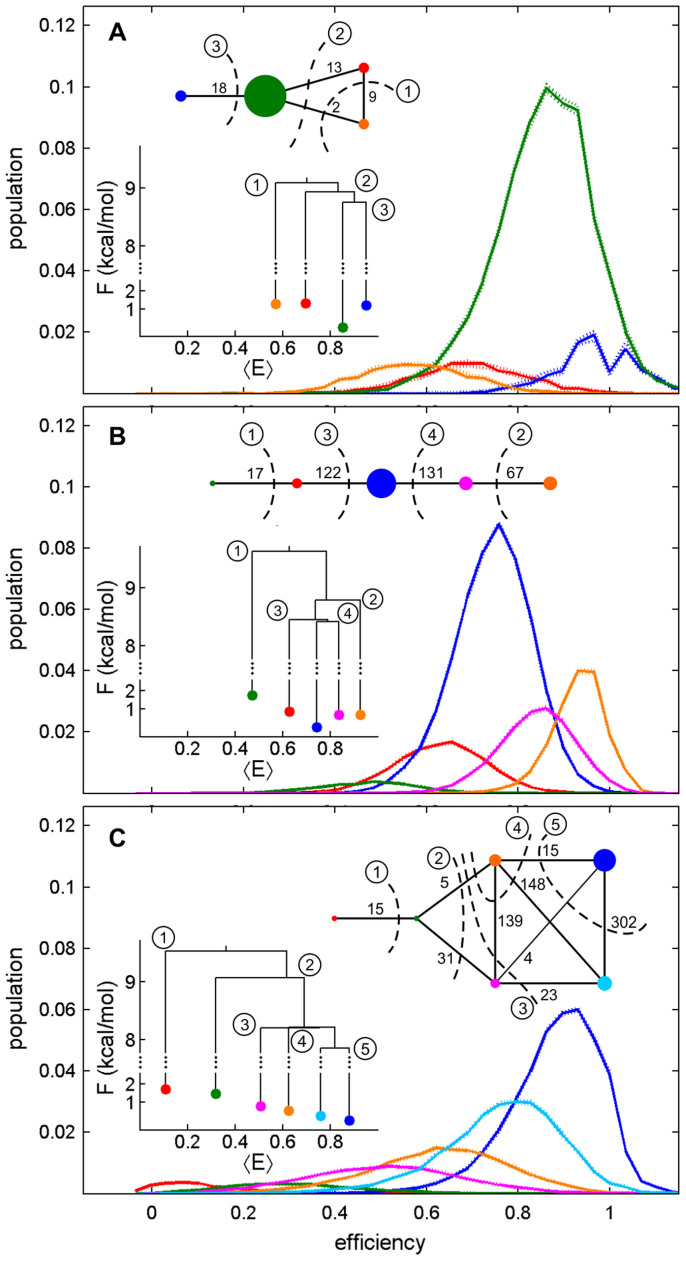
Results for the AMPA ABDs showing state distributions, state space networks, and TRDGs for the (A) glutamate-bound ABDs, (B) nitrowillardiine-bound ABDs, and (C) the UBP282-bound ABDs. Sizes of the nodes in the networks are proportional to the population of each state with the exception that all states having population < 10% of the most populous state have the same size. Each edge in the networks is labeled with the number of transitions along the edge. The colors of all nodes correspond to the colors of the state distributions in each panel. The networks and the TRDGs are labeled with circled numbers indicating the sequence of dividing surfaces used to construct the TRDGs.

Fig. 2 displays the SCISM-extracted state distributions with a segment length of 99 ms. Each segment contains 33 uniform time bins of length 3 ms. Segment lengths were chosen such that they contain a large enough number of samples to minimize sampling error, but are short enough such that they capture the time scale of the dynamics previously observed in the glutamate system^42^. The steady-state networks shown in Fig. 2 include the symmetrized number of transitions between each state in the network, and the cuts (i.e., dividing surfaces) used to construct the TRDGs for the experimental AMPA receptor systems; the overall transition rates across each of the dividing surfaces increase as the order (circled numbers) of the cuts increases. Various properties of the extracted states, including escape times, mean efficiencies, populations, free energies, and single-exponential behavior are shown in Table 1. Survival curves and escape time distributions are provided in SI Section 4, and full descriptions of escape time calculation, as well as network and TRDG construction are included in Methods. Barrier heights, i.e., the energy at the dividing surface between two (sets of) states, among the branches in the TRDGs of the glutamate-bound, nitrowillardiine-bound, and UBP282-bound ABDs are summarized in Table 2, where the columns ‘Branch 1’ and ‘Branch 2’ contain the mean efficiencies of the states on either side of the particular branch in the TRDG. Here, free energies at barriers linking the two branches were estimated via Eq. 10, and the barrier heights listed in the table are the differences between free energy at each barrier linking two branches and that of the lowest free energy state in each system.

Analysis of the glutamate condition^42^ via SCISM returned 4 states as shown in Fig. 2A. The most dominant state has a 74% occupation probability and a mean efficiency of 0.85, corresponding to an interdye distance of ~38 Å. A higher efficiency state has an occupation probability of 10%, resulting in nearly 85% occupation of states with mean efficiencies high enough to be considered a closed-cleft conformation^48^. Escape times indicate slower transitions from the high efficiency states to the lower efficiency states and faster transitions in the reverse direction. In addition, the TRDG suggests the lowest barrier for transition to be between the two lowest energy conformations at high efficiency. Overall, while there are conformational dynamics observed within the glutamate-bound ABD, these results suggest a relatively stable and closed ABD when bound to the full agonist glutamate.

Results for the nitrowillardiine-bound ABD^34^ are shown in Fig. 2B. A total of 5 states were returned for this system, with the most populated state having 〈*E*〉 = 0.74 and an occupation probability of 52%. Transitions within this system were slower in general when compared to the glutamate results, with escape times ranging from 200–800 ms. Non-single exponential behavior, determined by *χ*^2^ tests between extracted and fitted survival curves, was observed in the majority of the survival curves (see SI Figs. S9–S11 and Table 1), however, indicating that underlying states remain hidden in the noisy trajectories. Furthermore, the TRDG suggests the barrier heights of the landscape to be smaller in magnitude than barrier heights observed in the glutamate system, indicating a more active environment within the nitrowillardiine-bound ABD when compared to the glutamate-bound ABD.

Lastly, we discuss the results shown in Fig. 2C for the ABD when bound to the antagonist UBP-282^34^. SCISM returned 6 states for the antagonist-bound form, indicating a wider interdye range for the ABD than is observed for the full or partial agonist-bound ABD. While there is higher relative occupation at lower efficiencies, the most populous state (41%) has a high 〈*E*〉 of 0.88, which is well within the ‘closed cleft’ range provided in Ref. 34. However, when the TRDG and escape times are examined, the reason for the lack of channel activation in the antagonist-bound ABD becomes clear. The TRDG indicates that the antagonist-bound form can transverse the four states having FRET efficiencies 0.51, 0.62, 0.76, and 0.88 with barrier heights that are ~1 kcal/mol smaller than those found in the glutamate and nitrowillardiine results. The escape times are in the range of 200–500 ms, and while not significantly shorter than the full and partial agonist results, the most populous state (〈*E*〉 = 0.88) exhibits non-single exponential behavior, again an indication of the existence of further underlying states. Note in that the third and fourth dividing surfaces of the nitrowillardiine- and UBP-282-bound ABDs have almost same free energy barriers, causing uncertainty in the branching structures, which may reflect a more frustrated nature of the landscape than the glutamate-bound ABD. Taken together, these results point to a conformationally active ABD when bound to the antagonist. Not only are there more conformational states, but transitions out of conformations that are presumed to activate the ion channel are faster. This frustrated feature on the energy landscape coupled with the faster dynamics are the root of its antagonism.

In a broader sense, these results combine to paint a clearer picture of agonism and channel activation of AMPA receptors. When bound to the ABD, full agonist glutamate yields a stable and largely static closed cleft ABD through strong interactions both lobes. The interaction of the ABD with the partial agonist nitrowillardiine is weaker and/or sterically distorted, yielding an ABD cleft that is less closed and more active than that of the glutamate-bound form. Lastly, the weaker interactions between the ABD and the antagonist UBP-282 result in an even more active ABD that converts among various open and closed cleft conformations on a fast time scale. Overall, the interpretations provided by SCISM support the conjecture that channel activation is governed not simply by the degree of cleft closure, as the antagonist-bound ABD is closed more often than it is open, but by the agonist's ability to hold the cleft closed through strong interactions with the lobes of the ABD.

**Figure 3:**
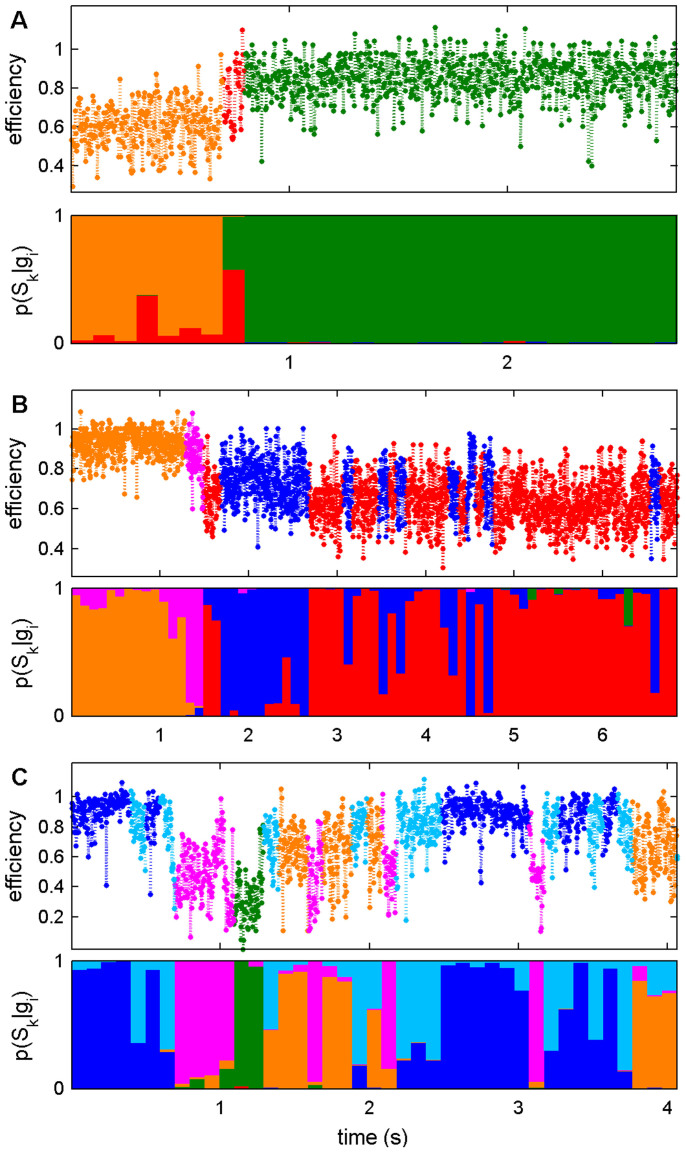
Representative trajectories of the AMPA ABDs for the (A) glutamate-bound, (B) nitrowillardiine-bound, and (C) UBP282-bound conditions. Segments in the upper sub-panels are colored with the color of their most probable states. Bar heights in the lower sub-panels correspond to the magnitude of the *p*(*S_k_*|*g_i_*). All colors correspond to those used in Figure 2.

This inference is further illustrated by the representative trajectories for each of the glutamate-bound, nitrowillardiine-bound, and UBP282-bound ABDs shown in Fig. 3. Each panel in Fig. 3 contains 2 sub-panels, with the upper sub-panel showing the representative trajectory, with each data point in each segment being colored according to the most probable state at the segment according to *p*(*S_k_*|*g_i_*), and the lower sub-panel illustrating the conditional probabilities *p*(*S_k_*|*g_i_*) of each state at each segment. Note that the colors of all states in Fig. 3 correspond to those used in Fig. 2. The data points within each segment in the upper panels are colored according to the most probable state, and the bar heights in the lower panels correspond to the magnitudes of the probabilities *p*(*S_k_*|*g_i_*) for each state at each segment.

Fig. 3A shows a glutamate-bound trajectory, Fig. 3B a nitrowillardiine-bound trajectory, and Fig. 3C an antagonist-bound trajectory. The trajectories in Fig. 3 clearly show the dynamical variability among the different conditions. In the upper panels, the most probable state sequences show that state-to-state fluctuations increase markedly from the glutamate-bound to the nitrowillardiine-bound and antagonist-bound ABDs. The difference in the uncertainty in state assignments shown in the lower panels also arises from the topographical features of the underlying energy landscapes. In the case of glutamate-bound ABDs, because of well-separated states with relatively large barriers (see Fig. 2 and Table 2), except around the transition region at 0.7–0.8 s, the identification of states does not change or fluctuate significantly. In contrast, ABDs bound with the partial-agonist and antagonist (Figs. 3B and 3C, respectively) show that the system belongs to multiple states along the time course due to relatively lower barrier heights (and of course experimental errors). This consequence is consistent with the topography of the energy landscape found in the TRDGs. For example, the states depicted by blue and light blue colors that are separated by the lowest barrier height in Fig. 2C tend to be those to which the system is multiply assigned along the time course (e.g., 0–0.7 s, 2.2–3 s in Fig. 3C), but the system is approximately assigned to a single state depicted by green color in 1.1–1.3 s in Fig. 3C, which is well separated by the larger barriers in Fig. 2C.

### Discussion

We have combined the information theoretical rate-distortion theory^38^,^39^ with the segmentation procedure of local equilibrium state analysis^12^, resulting in a new method to construct steady-state networks and extract effective free energy landscapes from noisy, experimental SM time-series. Through the incorporation of error into the procedure, we have developed a method to naturally extract the appropriate number of states by quantifying the contributions of experimental and finite sampling errors. Our method avoids assumption of this quantity as well as any assumptions regarding the properties of the states, such as the shapes of their distributions and their connectivity through the network. Furthermore, overfitting is naturally avoided by defining the level at which measurement errors and finite sampling errors dominate the data, thus eliminating the deleterious effects of overfitting and using the maximum amount of information contained within the data at the time scale of the segment length.

We demonstrated the new method to be successful in identifying the states underlying two simulated systems. An overdamped Langevin diffusion simulation on a 1-dimensional potential of mean force was used to test the method's proficiency at a broad range of signal-to-noise ratios. States and their properties were accurately extracted at reasonable experimental signal-to-noise ratios, establishing guidelines for the use of the SCISM procedure on empirical data in terms of the error magnitude. These results also confirm that the model selection procedure, which uses the magnitude of error contributions as a guide in determining the maximum number of states the data will allow to be extracted, ensures that overfitting does not occur, as the method returns fewer than the true number of states at low signal-to-noise ratios. A photon-by-photon simulation emulating a SM FRET experiment (Section S3 in the SI) was used to validate SCISM application to such experiments. Not only does the SCISM method accurately identify the correct number of states and their underlying distributions to greater than 95% overlap with the true distributions, but it also accurately identifies the state-to-state kinetics as well, as all extracted state lifetimes were in agreement within error of the true values.

We also applied the method to the experimental SM FRET data acquired for isolated agonist binding domains of the AMPA receptor GluA2 while bound to a full agonist, a partial agonist, and an antagonist. Our method uncovers new information on hierarchical organization of states buried in the experimental trajectories that deepens our understanding of the ion channel's mechanism of activation. Specifically, the results for the full agonist glutamate suggest a closed, stable, and largely static ABD. Stability decreases and the cleft distance increases when the ABD is bound to the partial agonist nitrowillardiine, suggesting that the ABD's interaction with the partial agonist is weaker and/or sterically distorted. This trend continues with the results for the antagonist. Although the results for this system indicate that the antagonist-bound ABD often populates a closed cleft conformation, transitions out of this conformation are fast. Taken together, our results for these experimental systems support the theoretical conjecture that the activation of the GluA2 receptor is not solely a product of the degree of cleft closure, but is also affected by the agonist's ability to hold the cleft closed in a stable fashion.

Our method bridges single molecule biology, energy landscapes, and complex networks into a single platform that starts from experimental data that are subject to experimental errors and finite sampling effects. It is thus expected to have an immediate impact in the SM community, aiding in the interpretations provided by SM experiments that take the multiple pathways through the entire steady-state network into account. Our method is unique in that, while such analyses are often hindered by experimental noise and finite sampling effects, SCISM turns these difficulties to its advantage, using them to select the appropriate number of states and avoid overfitting the system, and to extract the underlying states directly along the observed time-series. Although detailed balance among all states in all networks studied in this paper was verified (see the SI), the escape kinetics from some states were found to not follow single exponential behavior. In addition to the existence of multiple states as described above, this may indicate the possibility of heterogeneous, non-Markovian nature of the networks^10^,^19^,^47^, especially for the partial agonist-bound AMPA ABD experiment. Future work will focus on the identification of such behavior and the incorporation of these enhancements.

## Methods

### Details of the SCISM procedure

Clustering the set of segments ***g*** into the set of states ***S*** may take two general forms. The segments may either be forced to exist in only one cluster and no others, i.e., hard clustering, or they may be allowed to span multiple clusters, i.e., soft clustering. Soft clustering makes use of a conditional probability, *p*(*S_k_*|*g_i_*), of a state *S_k_* given the observation of a segment *g_i_*. Note that in terms of the conditional probabilities, hard clustering is simply a limit of soft clustering where all *p*(*S_k_*|*g_i_*) approach 0 or 1. The use of soft clustering is advantageous considering the noisy nature of SM FRET data, as the *p*(*S_k_*|*g_i_*) reflect uncertainties in state assignments of the segments.

The objective of rate-distortion theory is to minimize the amount of information needed to describe the set ***g*** in terms of the set of states ***S*** while maintaining a desired level of distortion. This information is 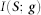, which, in information theoretical terms, is the average number of bits required to specify each segment 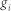 within the set of states ***S***.
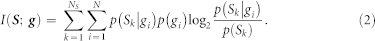
Minimization of 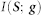 is accomplished via the method of Lagrange multipliers, as the functional in Eq. 1 above is designed to minimize 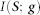 subject to the condition 〈*d*〉 ≤ *D* where *D* is some desired level of distortion. Here we have introduced *p*(*g_i_*), the probabilities of each of the segment pmfs *g_i_*and the probabilities *p*(*S_k_*) of each of the states *S_k_*. The conditionals *p*(*S_k_*|*g_i_*) are those discussed above, which are normalized across ***S*** for each *g_i_*. The average distortion across all states,
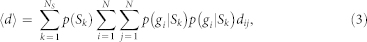
is the mean of the Kantorovich distances *d_ij_* for each pair of distributions *g_i_* and *g_j_* within the set of states ***S***.

Minimization of the functional in Eq. 1 is a well-known variational problem that involves setting the derivative of the functional, with respect to the variables *p*(*S_k_*|*g_i_*), to zero. Numerical values for the formal solution to this variational problem^39^,
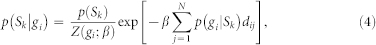
are obtained via an iterative procedure known as the Blahut-Arimoto algorithm^48^,^49^. Note that a normalization function, *Z*(*g_i_;β*), has been introduced in Eq. 4, and is given by
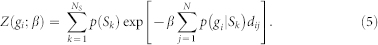
The probability of state *S_k_* is calculated via the conditionals *p*(*S_k_*|*g_i_*).


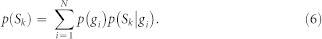
As is evident in the above Eqs. 4 and 6, the variables *p*(*S_k_*) and *p*(*S_k_*|*g_i_*) must be self-consistent, and this condition is met by alternating between calculation of the *p*(*S_k_*) and *p*(*S_k_*|*g_i_*) and iterating over both calculations until convergence in the functional value is reached. Practically, the matrix *p*(***S***|***g***) is randomly initialized, which may result in convergence to a local minimum, so the algorithm is initialized multiple times and the result minimizing the value of the functional is returned. This calculation returns the conditional probabilities *p*(*S_k_*|*g_i_*) for a particular number of states *N_S_* and a particular value of the Lagrange multiplier *β*.

For appropriate application to the noisy, finite time-series acquired in SM measurements, we must consider the contributions of error in the measurement as well as in the construction of the segment pmfs. Empirical error arises from various sources, including instrumental sources such as shot-noise and photophysical sources such as quantum yield fluctuations of the fluorophores. According to the central limit theorem, the collective contribution of these sources of error follows a normal distribution^30^. Empirical error is therefore incorporated by randomly sampling each original data point from a normal distribution whose mean is the value of the observable and whose standard deviation is the associated empirical error. Sampling error arising from finite sampling of the time-series is incorporated by bootstrapping, e.g., resampling the data points comprising the segments with replacement^50^. That is, for each time segment *g_i_*, we generate a possible realization by taking into account experimental errors and statistical fluctuation from finite sampling. Randomly sampled and bootstrapped segments are then used to construct a set of segment pmfs for each segment *g_i_*, and these pmfs are used to calculate the statistical uncertainty of pairwise distances *d_ij_* among all segments. The *d_ij_* are subsequently used to calculate a mean distortion (via Eq. 4) for a clustering result with a particular *N_S_* and *β*. Convergence of the bootstrapped distortion distribution is ensured by incrementally increasing the number of bootstraps, appending the new bootstraps to the existing ones, then using a two-sample, two-tailed Kolmogorov-Smirnov (K-S) hypothesis test to verify that the distribution does not change with the addition of new bootstraps. A supporting figure (Fig. S12) and an accompanying description are provided in the SI.

The remaining issue is to choose the number of states *N_S_* and *β,* i.e., the model to best describe the system under the elucidation of experimental errors and those from finite sampling. Selecting the simplest model that best fits the data without overfitting requires explicit definition of what is meant by the terms simplest, best fit, and overfit. The simplest model has the smallest value of *I*(***S****;****g***). For example, in the trivial case that there is only one state, *I*(***S****;****g***) vanishes. The simplest model, however, may give rise to large distortion. In this sense, distortion is a measure of the quality of the model's fit to the data. The model that best fits the data will have the lowest level of distortion. This is the oppositely trivial case in which each segment belongs to its own cluster and to no others, yielding zero distortion. Although intra-segment distortions (i.e., distance between a pmf of segment *g_i_* and that of itself, or self-distortion) vanish in the absence of error, nonzero self-distortion arises from empirical and sampling errors. Distortion is therefore present within even the best fitting model, which brings us to overfitting. The incorporation of error provides a simple and natural way to avoid overfitting by defining the level of distortion that is present within the best fitting model; that is, defining the amount of distortion due to error in the data. If the mean distortion of a particular model falls within the range of distortion due to error, then the distortion arising within the model can be attributed to error, and thus the model attains the minimum *achievable* level of distortion. Further increasing the model's complexity constitutes overfitting, as there is no further information available at the time scale of the segment length.

Distortion due to error is estimated by bootstrapping the intra-segment distortions in the same manner as described above, and is further detailed in the SI. A confidence interval, e.g., 95%, on the self-distortion distribution is selected, thus providing a ‘distortion cutoff’ and a means to select the appropriate model. The SCISM algorithm is initialized at small *β* for each *N_s_* and is incrementally increased either until the distortion cutoff is passed or until the mean distortion distributions are unchanged (*via* K-S test) with further increase in *β*. Models that satisfy the distortion cutoff are compared directly via *I*(***S****;****g***), and the model with the smallest *I*(***S****;****g***) satisfying the distortion cutoff is selected as the appropriate model. Further details are illustrated in Fig. S2 describing the results of the overdamped Langevin simulation. Once the model has been selected and the states obtained, we construct steady-state networks, calculate escape times, construct TRDGs, and infer all of their accompanying error estimations.

### TRDG Construction

Effective free energy landscapes, illustrated as transition disconnectivity graphs (TRDGs) as shown in Fig. 2, are constructed from the network of the states' residence and transition probabilities according to Krivov and Karplus^24^,^25^. In particular, let *N_ij_* be the number of transitions from the state *i* to the state *j*, and let the total number of observations be *N* = Σ*_i,j_N_ij_*. Then the relative free energy of the state *i* is
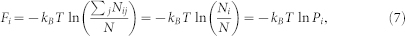
where *N_i_* is the number of visits to state *i*, *k_B_* is the Boltzmann constant and *T* is the temperature (298 K in this paper). The free energy at the barrier separating the state *i* and the state *j* denoted by *F_ij_* is calculated as follows: suppose that the rate constant from the state *i* to the state *j*, *k_ij_*, is represented by Kramers' rate theory, i.e., *k_ij_* ≈*τ*_0_^−1^ exp(−(*F_ij_*−*F_i_*)/*k_B_T*) where the pre-exponential factor *τ*_0_ roughly corresponds to the decay timescale of the autocorrelation function for motion exerted by friction from the environment. In this paper, we choose *τ*_0_ to be 1 μs as for the typical timescale of the fastest protein folding^51^,^52^ for the sake of simplicity. Then, the following relation holds.
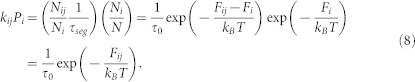
Here, *τ_seg_* is the observation time, i.e., the length of a segment in the original time-series. Then, *F_ij_* is elucidated by
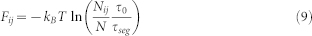
Note that, in order to validate the concept of the free energy at the barrier separating the state *i* and the state *j,* the condition *F_ij_* = *F_ji_* needs to hold, implying the requirement of detailed balance *k_ij_P_i_* = *k_ji_P_j_*.

The branching structure of the TRDGs arises via the application of the max-flow, min-cut protocol detailed by Krivov and Karplus^24^,^25^ to the state-to-state network. Free energies at a series of barriers (i.e., cuts, dividing surfaces) linking two disjoint sets *I* and *J* of the network in TRDG are elucidated via Eq. 9 in which the number of transitions per unit time *τ_seg_* between the states *i* and *j*, *N_ij_*, is replaced by the number of transitions across the cut. That is,
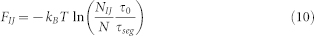
where 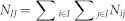. It is of note that the transition matrix must be symmetrized in order to apply this protocol. To ensure that the symmetrization of the transition matrix does not invalidate the resulting TRDG and the concept of the free energy landscape, detailed balance among the states in the steady-state network must be verified. Specifically, detailed balance requires the numbers of forward and backward transitions to be equivalent, i.e., *N_ij_ = N_ji_* for all pairs of states *i* and *j*. Considering, however, that single-molecule measurements return time-series of finite length, this condition may not necessarily hold. We thus performed hypothesis tests under the null hypothesis that detailed balance holds as described in the SI. All tests suggested that the detailed balance hypothesis cannot be rejected for systems reported here.

## Author Contributions

J.N.T. and C.B.L. contributed equally to this work. J.N.T. analyzed the data and wrote the algorithms. J.N.T. and T.K. wrote and prepared the manuscript. C.B.L. and T.K. supervised the research. D.R.C. performed the single-molecule experiments, and C.F.L. supervised the experimental research. All authors discussed the results and implications of the research and commented on the manuscript at all stages.

## Supplementary Material

Supplementary Information Supporting Information for Error-based Extraction of States and Energy Landscapes from Experimental Single-Molecule Time-Series

## Figures and Tables

**Table 1:** Various state properties returned for the AMPA ABDs, including mean efficiencies, occupation probabilities, free energies, escape times with 95% confidence intervals, and χ^2^ p-values for single-exponential behavior

〈*E*〉	*P*(*S_k_*) (%)	*F_i_* (kcal/mol)	escape time (ms)	χ^2^ p-value
**Glutamate**				
0.97	10	1.21	308 (220,425)	0
0.85	74	0	674 (589,753)	1
0.75	8	1.31	185 (145,220)	1
0.64	8	1.29	310 (240,384)	1
**Nitrowillardiine**				
0.93	16	0.68	807 (690,928)	0
0.84	17	0.68	288 (260,300)	0
0.74	52	0	664 (618,698)	0
0.66	12	0.88	290 (260,308)	0
0.47	3	1.75	502 (388,634)	1
**UBP282 Antagonist**				
0.88	41	0	490 (458,519)	0
0.76	27	0.26	223 (207,236)	1
0.62	16	0.56	207 (193,217)	1
0.51	11	0.78	220 (203,236)	1
0.32	3	1.47	250 (217,283)	1
0.11	2	1.71	512 (420,619)	0

**Table 2:** Free energy barriers for the TRDGs of the AMPA ABDs. States are denoted by their mean efficiencies. Barrier heights are measured relative to the lowest energy state for each system

Branch 1	Branch 2	Barrier height (kcal/mol)
**Glutamate**		
0.58	0.69, 0.85, 0.97	9.05
0.69	0.85, 0.97	8.95
0.85	0.97	8.75
**Nitrowillardiine**		
0.47	0.63, 0.74, 0.84, 0.93	9.59
0.93	0.63, 0.74, 0.84	8.79
0.63	0.74, 0.84	8.45
0.74	0.84	8.42
**UBP282 antagonist**		
0.11	0.32, 0.51, 0.62, 0.76, 0.88	8.54
0.32	0.51, 0.62, 0.76, 0.88	9.07
0.51	0.62, 0.76, 0.88	8.22
0.62	0.76, 0.88	8.21
0.76	0.88	7.85
