# Supplementary material for: Error-based Extraction of States and Energy Landscapes from Experimental Single-Molecule Time-Series
Source: Sci Rep. 2015 Mar 17;5:9174. doi: 10.1038/srep09174 (PMC4361849; doi:10.1038/srep09174)
Supplement: Supplementary Information — Supporting Information for Error-based Extraction of States and Energy Landscapes from Experimental Single-Molecule Time-Series [file srep09174-s1.pdf]

Error-based Extraction of States and Energy Landscapes from Experimental Single-Molecule Time-Series

J.N. Taylor[†], Chun-Biu Li[†], David R. Cooper[‡], Christy F. Landes[‡], and Tamiki Komatsuzaki[†]*

[†] *Research Institute for Electronic Science, Hokkaido University, Kita 20 Nishi 10, Kita-Ku, Sapporo 001-0020 Japan*

[‡] *Department of Chemistry, Rice University, P.O. Box 1892, Houston, TX 77005*

* to whom correspondence should be addressed: tamiki@es.hokudai.ac.jp

Supplementary Information

Section S1: The performance of SCISM vs. Signal-to-Noise Ratio

In this section we assess the performance of the SCISM procedure for a broad range of signal-to-noise ratios (SNRs). We perform this analysis on a simulated system with known properties, using a one-dimensional Langevin system in the high friction limit. We simulate diffusion on the potential of mean force $V(x)$,

$$V(x) = \sum_{i=1}^{5} V_i(x) = \sum_{i=1}^{5} A_i \exp\left[-\frac{(x - x_i)^2}{2\sigma^2}\right], \tag{S1}$$

shown along the coordinate x in Fig. S1A. Here the centers x_i and depths A_i of each well are set to be 0.2δ, 0.35δ, 0.5δ, 0.65δ, 0.8δ, and -8ε,-7ε,-6ε,-9ε,-6ε, for i = 1,2,3,4,5, respectively, with $\sigma = 0.04\,\delta$ used for all wells. The coordinate x is non-negative and normalized by a value δ, and the well depths A_i are multiplied by the unit of energy ε. Trajectories were simulated using overdamped Langevin dynamics,

$$\gamma \frac{dx}{dt} = -\frac{dV}{dx} + \xi(t). \tag{S2}$$

Here γ is the friction constant, and $\xi(t)$ denotes the random force exerted by the environment, which is computed as $\sqrt{2k_BT/\gamma} \cdot N(0, dt)$ with $N(0, dt)$ being real numbers that are randomly sampled from a normal distribution having zero mean and standard deviation equal to the time step, dt. The temperature T is set so that thermal energy $k_BT = 0.6\varepsilon$ is much smaller than any of barrier heights, where k_B is the Boltzmann constant. The friction constant γ is chosen from $D = k_BT/\gamma$ so that diffusion constant D is approximately $0.2\delta^2$/s. Well depths A_i and widths σ are chosen so that the relaxation times inside the wells $\lambda^{-1} = \gamma/\omega^2$, where ω^2 is the mean curvature of the potential of mean force at the centers of the wells, are approximately 18 ms, and are much longer than the chosen time step dt = 1 ms. The well-to-well transition time scales for the system are in the range of approximately 200-900 ms, which are much longer than the relaxation times inside the wells. Trajectory lengths are chosen stochastically from an exponential distribution with mean 2.5 s (see Eq. S5 below), resulting in 100 trajectories with a mean length of 2.66 s in 1 ms time bins. Segmentation with a segment length of 100 ms (100 time steps per segment) results in 2609 segments that are input to the SCISM procedure.

The simulated system is summarized in Fig. S1. Trajectories simulated *via* Eq. S1 on the potential of mean force $V(x)$ (Fig. S1A) resulted in the Boltzmann distributions for each state, i.e., distributions along the coordinate x within each well $V_i(x)$, shown in Fig. S1B, where colors are used to visually

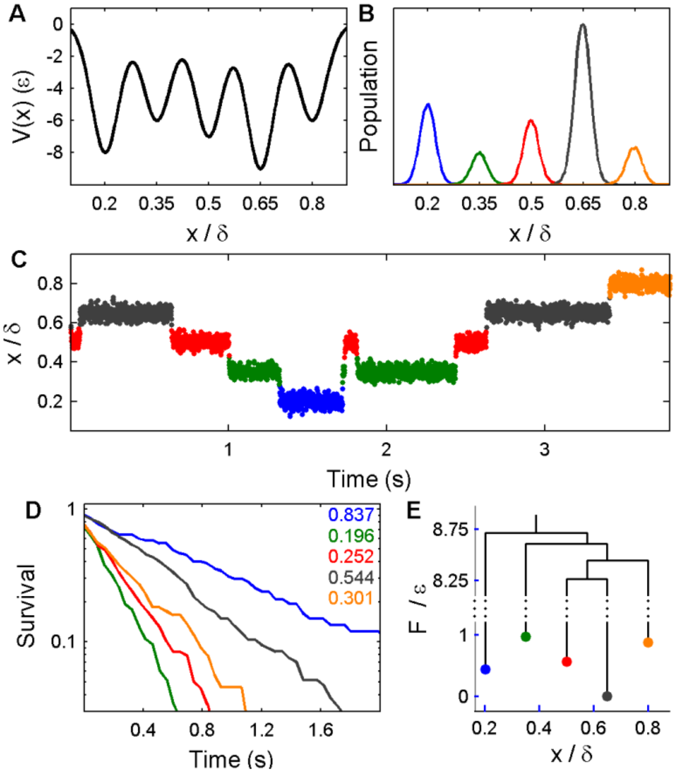

Figure S1. Properties of the 1-dimensional diffusion simulation. **A)** The potential of mean force. **B)** the error-free state distributions. **C)** A sample trajectory colored by state residence. Colors correspond to **B**. **D)** Survival curves and escape times for the 5 states. **E)** the true TRDG for the system.

differentiate each state. A representative trajectory is shown in Fig. S1C, with color along the time axis corresponding to the color of the state distributions in Fig. S1B. Survival probability curves and mean escape times are shown in Fig. S1D in corresponding color. Finally, the TRDG extracted from the error-free system is shown in Fig. S1E. Constructing the TRDG required calculating transition probabilities among states. To count the transitions, state identities were partitioned at the top of each barrier. Barrier crossings qualified as transitions only if the system reached an energy whose value is lower than the barrier height in question by the thermal energy $k_B T$. For example, a barrier crossing at an energy near the barrier height followed by an immediate return to the originating state was not counted as a transition.

To quantify the effects of experimental errors and establish limitations in the application of the SCISM procedure, normally distributed errors of various magnitudes were sampled into the error-free simulated trajectories. We note here that, although the simulated errors are normally distributed, the SCISM procedure itself does not rely on the functional form of the noise. In the case that the error distributions are known, regardless of whether they are Gaussian or non-Gaussian, they can be easily accounted for within the confines of our method by resampling the data from the known error distributions. In the case they are unknown, control experiments in which the value of the observable is fixed to a constant value with a model system can be used along with bootstrapping techniques to estimate the error distributions.

Specifically, we incorporated error magnitudes Δ of 0.025, 0.05, 0.1, 0.15, 0.2, 0.25, and 0.3 along the coordinate x. For a FRET or fluorescence experiment, these error magnitudes correspond to mean SNRs of 20, 10, 5, 3.3, 2.5, 2, and 1.67, respectively.

We begin with model selection in the case of the $\Delta = 0.1$ error magnitude. Model selection uses the mean distortion, which is the mean Kantorovich distance among all segments and states as described in Eq. 3 of the main text, to assess the quality of the model's fit to the data. Considering that grouping dissimilar segments into the same state will result in a higher level of distortion, a model's mean distortion serves as a quality of fit measure in the sense that models that fit well to the data will minimize distortion, and models that do not fit well to the data will have larger distortion. In the error-free case the mean distortion of a good fitting model will approach zero. However, in the presence of error nonzero distortion arises from the error. Estimation of the level of distortion arising from error provides a measure by which the level of model complexity at which overfitting begins to occur can be determined. If the mean distortion of a particular model falls within the range of distortion due to error, then the distortion arising within the model can be attributed to error, and thus the model attains the minimum *achievable* level of distortion. Fig S2A shows a representative trajectory from the simulated data set, from which we use the segment highlighted in red to demonstrate estimation of the distribution of distortion due to error.

Firstly, to incorporate sampling error arising from the finite sampling of the time series, the segment in question is bootstrapped, i.e., resampled with replacement. For example, a segment containing 100 samples is resampled with replacement 100 times to construct the bootstrapped segment. In addition to finite sampling error, we also have to incorporate measurement errors. Next, the each of the bootstrapped resamples is randomly sampled from its empirical error distribution, e.g., in this demonstration, instead of using the resampled data point x_r, we randomly resample from $N(x_r,\Delta_r)$, where $\Delta_r = 0.1$ is the error magnitude for this particular x_r. This produces a set of data points for the segment in which possible effects of empirical and sampling errors are manifested. This process is then repeated, resulting in two representations of the segment probability mass function (pmf), as shown in Fig. S2B. In Fig. S2C, the Kantorovich distance – the area of the shaded region between the two cumulative distribution functions (cdfs) – between the two differing representations of the segment pmf is shown to be nonzero. In contrast, if no measurement or finite sampling errors are present, then this distance should be zero. This process is repeated 500 times, producing the distribution of distortion due to error for this particular segment as shown in Fig. S2D. Convergence of this distribution was ensured via with a 2-sample Kolmogorov Smirnov tests. Although convergence is typically achieved between 50 and 100 samples for each segment, 500 samples are produced for each segment to ensure sufficient sampling. Performing the operation on all segments in all trajectories produces the overall distribution of distortion due to error, which is displayed in Fig. S2E. We then choose a confidence limit, in this case, the 95% confidence limit, yielding a "distortion cutoff," or the level at which distortion arising from the quality of a model's fit cannot be distinguished from distortion arising from error. Models that satisfy this distortion criterion are displayed in red in Fig S2F, which plots mean distortion versus the number of states N_s and the value of the Lagrange multiplier β, while those that do not are shown in black.

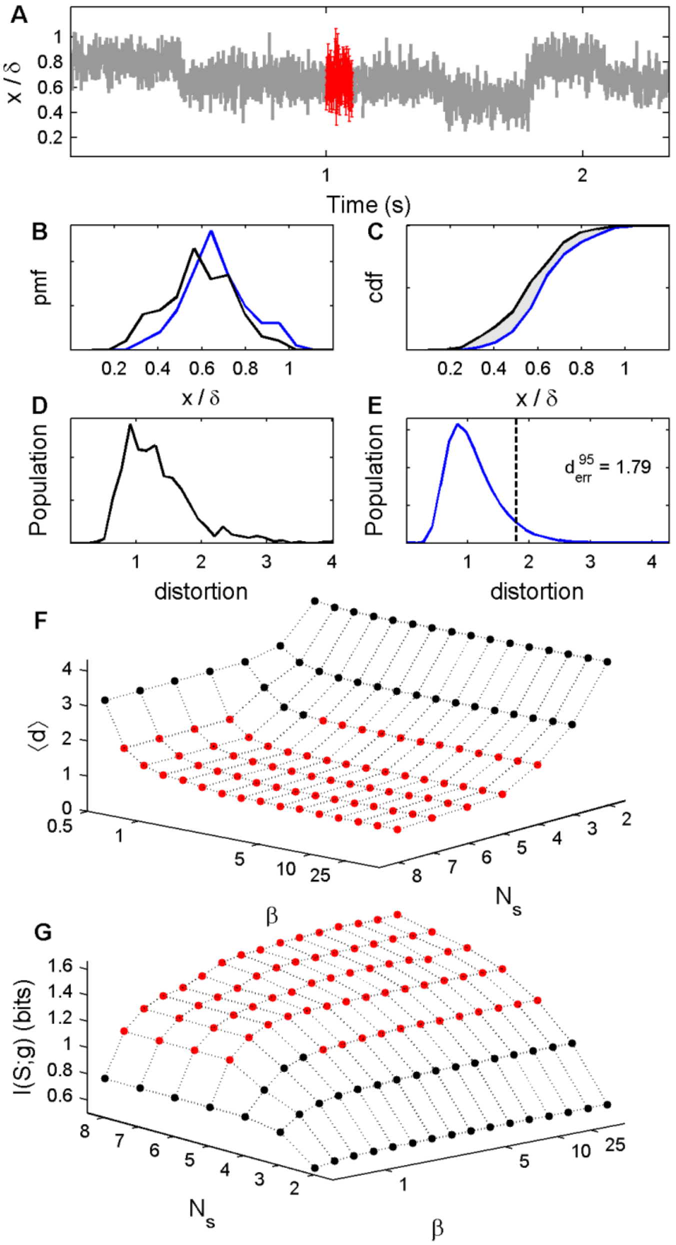

Figure S2. Model selection in the SCISM procedure. **A)** a representative trajectory from the simulated data set with error magnitude of 0.1. **B)** Two realizations of the segment pmf for the segment highlited in red in **A**. **C)** cdfs corresponding to the pmfs in **B** showing nonzero Kantorovich distance. D) The distribution of distortion due to error for the highlighted segment. **E)** Distribution of distortion due to error for all segments in all trajectories showing the 95% confidence limit on the distortion due to error. **F)** Mean distortion versus *N*s and β. **G)** Mutual information versus *N*s and β. Models satisfying the distortion criterion and shown in red in **F** and **G**, while those that do not are shown in black.

Now that the subset of models achieving the minimum level of distortion has been identified, we must select the appropriate model for further analysis. For this we examine the values of the mutual information $I(\boldsymbol{S};\boldsymbol{g})$. This quantity is the average amount of information in bits required to specify a segment within the context of a particular model. That is, the mutual information is a direct measure of a model's complexity, as more complex models will require more information on average to describe a particular segment. In the same spirit as other model selection methods such as the Bayesian Information Criteria and Minimum Description Length principle, in which more complex models are penalized according to their complexity, we wish to minimize the amount of information required to describe the data set within the context of the model while maintaining a high quality of fit. From Fig. S2G, we identify this model as having five states and a value of β of approximately 0.75. We note here that, although the model having N_s = 4 and β ~ 1.25 satisfies the distortion criterion, its value of $I(\boldsymbol{S};\boldsymbol{g})$ is larger than that of the selected model, thus it is a more complex model than the selected model despite having a fewer number of states.

The appropriate model having been selected, further analysis of the results for the error magnitude Δ = 0.1 are shown in Fig. S3. The SCISM procedure returned five states whose distributions (solid) are shown alongside the true distributions (dashed) in Fig. S3A. Overlap coefficients between the true and extracted distributions are also shown in corresponding color. These overlap coefficients O_i between the true distributions $P_i^{\text{true}}(x)$ and the extracted distributions $P_i^{\text{SCISM}}(x)$, defined by

$$O_i = \sum_x \min_x \left(P_i^{\text{true}}(x), P_i^{\text{SCISM}}(x)\right), \qquad (S3)$$

represent the amount of overlap between the true and extracted probability distributions for each state. As shown in Fig. S3A, all state distributions are extracted accurately, having overlap coefficients greater than 0.95. A representative trajectory is shown in Fig. S3B with colors indicating true state residencies. Colors correspond to those of the distributions in Fig S3A. Fig. S3C shows the same trajectory colored according to the maximum probability sequence returned by SCISM, i.e., each segment g_i is colored according to the state S_k having $\max_k\ p(S_k|g_i)$. The bar heights in Fig. S3D correspond to the magnitude of each conditional probability $p(S_k|g_i)$ at each segment. It is of note that the residence in the state having mean 0.35 near 0.4 s (shown in grey) in the trajectory is shorter than the segment length of 100 ms, and its assignment is therefore absent in the maximum probability sequence. The conditional probability of this state at this segment, however, is significant, and thereby demonstrates the power of SCISM in state assignment. Indeed, even though this state residence is shorter than the segment length, the assignment of probability of this state to this segment will manifest its occurrence in the state distribution, the escape time, and the TRDG. The extracted TRDG in Fig S3E is shown alongside the true TRDG in Fig. S3F. Comparison of the true and extracted TRDGs reveals good agreement, as the free energies of all states are returned accurately. The branching structure of the TRDGs and barrier heights are also accurately returned, as they are within the error boundaries of the extracted barrier heights.

Overall, Fig. S3 shows a complete and accurate characterization of the states, their dynamics, and the free energy landscape of this system with an error magnitude of 0.1. In the context of a FRET or fluorescence experiment, this error magnitude corresponds to the collection of an average of 25 photons

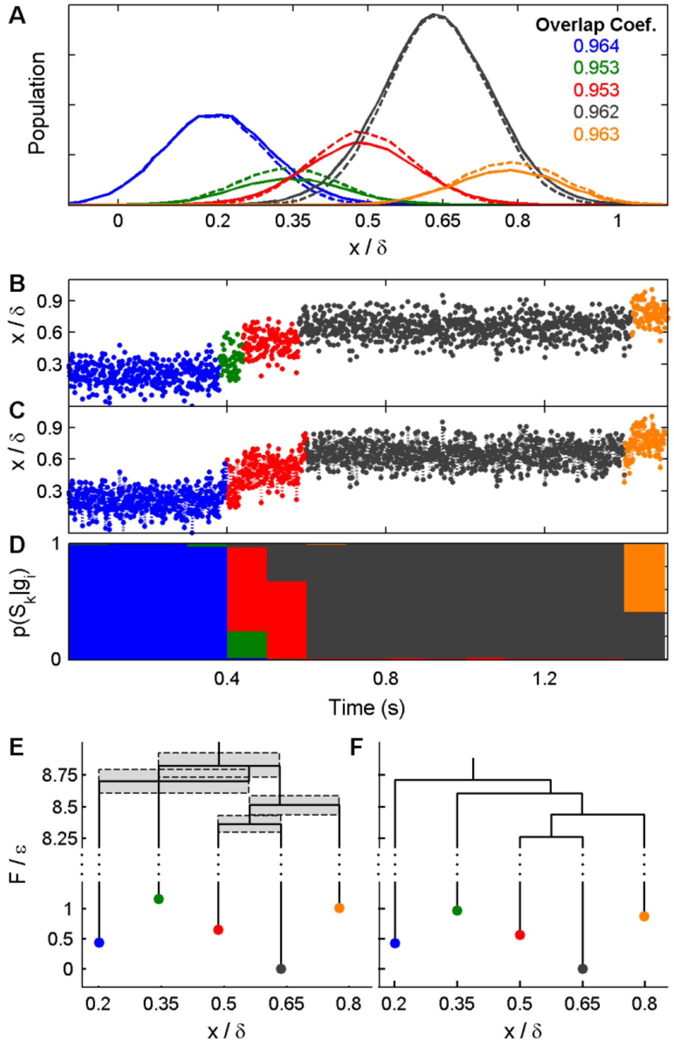

Figure S3. Results for the 0.1 error magnitude. **A)** Extracted state distributions (solid), true state distributions (dashed) and overlap coefficients. **B)** Trajectory colored by true state residences. **C)** Trajectory colored by maximum probability SCISM state assignments. **D)** Bar heights correspond to state probabilities at each segment along the trajectory. **E)** The SCISM-extracted TRDG. Grey boxes indicate 95% confidence intervals for the barrier heights. **F)** The true TRDG.

per time step and a signal-to-noise ratio of 5. In comparison to the average error magnitude of 0.08 efficiency units in the AMPA systems of the main text, the results returned for this simulated system demonstrate the proficiency of the SCISM procedure at similar levels of empirical error. It is of note that the extent of reproducibility may depend on each quantity; state identification is more robust than the estimation of barrier height and branching structure of TRDG. The estimation of barrier height and branching structure of TRDG requires kinetic information, that is, the accuracy in escape times and the appropriate choice of rate theory such as Kramers theory or Grote-Hynes theory are crucial.

As mentioned above, this analysis was performed for a range of error magnitudes in order to show the limitations of the SCISM procedure. Fig. S4 displays the results of the analysis at error magnitudes of

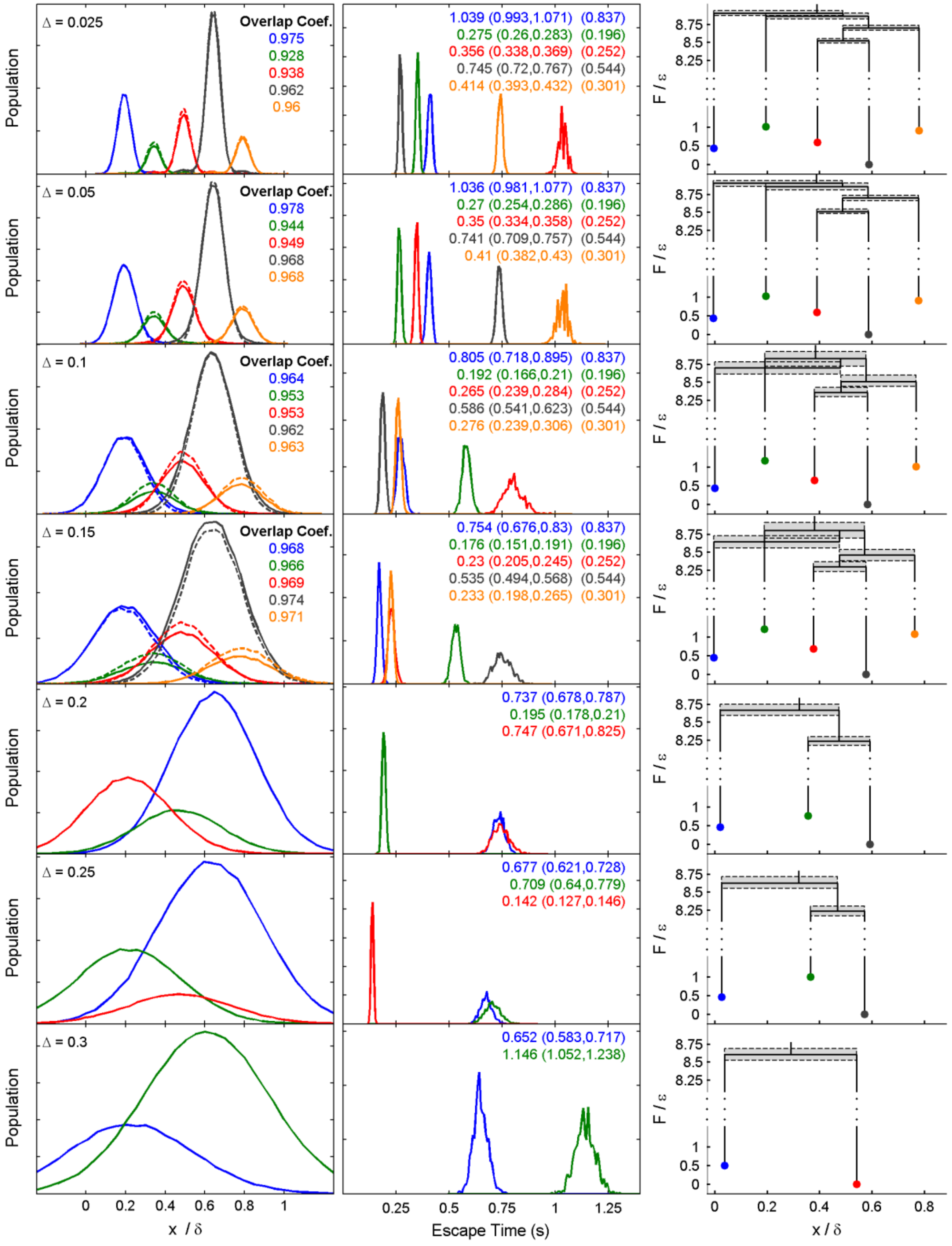

Figure S4. Comparison of SCISM-extracted state properties. Error magnitude increases from top to bottom. *left column:* The extracted state distributions (solid) with the true distributions (dashed) and overlap coefficients if the true and extracted numbers of states correspond. *center column:* Escape time distributions for each extracted state are shown with the most probable escape time and the 95% confidence limits. The true escape time is shown if the number of true and extracted states correspond. *right column:* Extracted TRDGs for each error magnitude. Grey boxes denote the 95% confidence intervals on each of the barrier heights.

0.025, 0.05, 0.1, 0.15, 0.2, 0.25, and 0.3. The left column of Fig. S4 shows the extracted state distributions (solid), as well as the true state distributions (dashed) and the overlap coefficients if the number of extracted states corresponded to the number of true states. The center column shows the escape time distributions for each extracted state, the most probable escape times with their 95% confidence limits, and the true escape times in parenthesis if the numbers of extracted and true states agree. The right column shows the TRDGs extracted at each error magnitude. All colors of all states correspond to those used in the previous figures. Fig. S5 shows state assignments for the same representative trajectory at each error magnitudes that returned the true number of states. Each subplot of Fig. S5 is labeled with its error magnitude, and contains three panels. The upper panel shows the trajectory colored by true state residencies, the center panel shows the maximum probability state assignments from the SCISM procedure, and the bar heights in the lower panel correspond to the magnitudes of the conditional probabilities $p(S_k|g_i)$ of each state at each segment.

As shown by Fig. S4, SCISM accurately returns the properties of the system for a wide range of error magnitudes. Specifically, for the error magnitudes of 0.025, 0.05, 0.1, and 0.15, corresponding to signal-to-noise ratios of 25, 10, 5, and 3.3 in a FRET or fluorescence experiment, our method returns the appropriate state distributions and free energies at individual states with a high degree of accuracy. Escape times coincide with the true values within 1 to 2 segment lengths, i.e., 100-200 ms, with the segment lengths being regarded as the time resolution in the SCISM procedure. In fact, when the segment length is chosen as 50 ms, the differences between the extracted and true escape times are approximately half of those with 100 ms segment lengths (not shown). Note that the escape times are comparably overestimated at higher signal-to-noise ratios, owing to decreased uncertainties in state assignments in combination with dynamics that are often faster than the segment lengths. This also manifests in the TRDGs, as the barrier heights are slightly overestimated as well. At lower signal-to-noise ratios of 5 and 3.3, we find better agreement in all state properties, including distributions, escape times, free energies and barrier heights within the error boundaries of the extracted barrier heights. This increased accuracy at higher noise level seems counterintuitive, but owes to the methodical treatment of the errors in the SCISM procedure, leading to more accurate, if less certain, state assignments. For example, the region of the trajectories shown in Fig. S5 near 1.75 s (segment range is indicated by vertical dashed lines) contains a residence in the state having mean of 0.35 (green) that is shorter than the segment length of 100 ms (whose). At higher signal-to-noise ratios, the decreased error leads to decreased uncertainty in the state assignments, i.e., the maximum conditional probability of the state assignment is nearer to unity than it is at lower signal-to-noise ratios. When generating the sequences to be used for transition probability and escape time calculations, this segment is less frequently assigned to the state having mean 0.35, leading to longer escape times in the maximum probability assignment, the state having mean 0.5. This effect is observed visually in the lower panels of Fig. S5, showing the magnitudes of the maximum conditional probabilities move away from unity as the error magnitude increases. For this reason, more accurate estimates of the escape times were obtained for these relatively low signal-to-noise levels.

Additionally, Fig. S4 establishes the limitations of the SCISM procedure in extracting the correct information from the data. As is obvious from Fig. S4, extracted results for error magnitudes that are

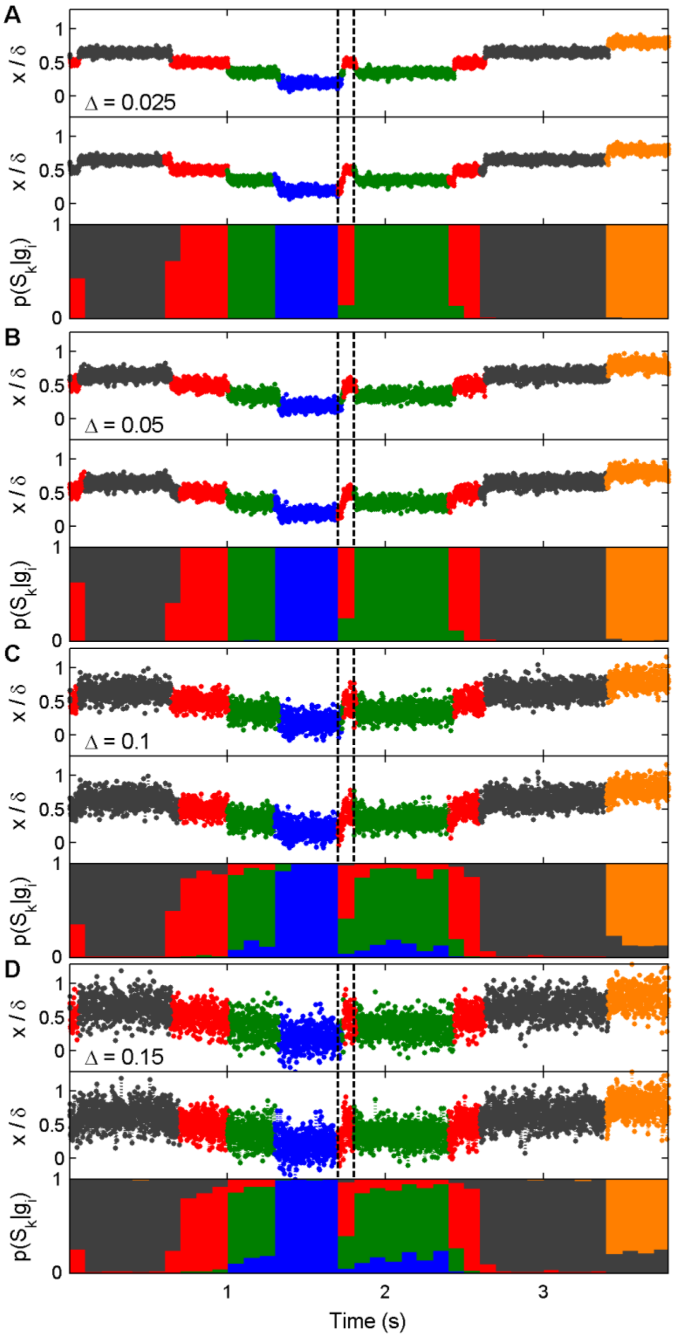

Figure S5. True and extracted state assignments and conditional probabilities for error magnitudes returning the true number of states. **A)** Error magnitude Δ = 0.025. **B)** Error magnitude Δ = 0.05. C) Error magnitude Δ = 0.1. **D)** Error magnitude Δ = 0.15. Each subplot contains 3 panels. The upper panel shows the trajectory at corresponding Δ with true state assignments, the center panel shows the SCISM-assigned states along the same trajectory, and the bar heights in the lower panel correspond to the magnitudes of the conditional probabilities $p(S_k|g_i)$.

larger than 0.15 do not contain the appropriate number of states. Considering that this error magnitude corresponds to a signal-to-noise ratio of ~2.5 for a FRET or fluorescence experiment, which means that an average of only 6 photons per time step were collected, this is to be expected. The data simply contain too much noise for the system to be properly characterized. The model selection procedure appropriately accounts for this fact however, as the extracted systems are less complex than the true system. In other words, this also verifies that our procedure does not overfit, i.e., extract more information than is justified by the data, at low signal-to-noise ratios. As the signal-to-noise ratio decreases further, the models become less and less complex, as shown in Fig. S4. Furthermore, decreasing the signal-to-noise ratio to less than 1.67 results in the identification of only one state (not shown), as the distortion due to error is larger than the mean distortion among the segment pmfs at these levels of error. Note however that the merging of states at higher noise levels provides some information concerning the underlying landscape. That is, states separated by lower barriers and/or having shallow well depths are not easily differentiated. Conversely, states that are separated by high barriers and/or having deep well depths are still identified, e.g., the states at $x = 0.2\delta$ and $x = 0.65\delta$ having the highest barriers and deepest well depths are the states that are recovered prior to collapsing into the single state, indicating that these states are separated from the other states with higher barriers, which is actually the case in this example. This is an advantage of the SCISM method, as the model selection procedure ensures the data are not overfit by accounting for the errors in the measurement.

Section S2: Empirical Errors with an Unknown Bias

Above, we demonstrated the performance of SCISM versus SNR in the case of normally distributed empirical errors. However, it is well known that not all empirical errors are normally distributed. Measurements errors may follow another type of distribution or may contain unknown contributions such as a systematic bias. As discussed above, known error distributions are easily accommodated by the SCISM procedure, whether they are normal or otherwise. But the nature and magnitude of systematic errors are often unknown. If the error distributions are assumed to be unbiased but are actually biased, how are the results of the SCISM procedure affected?

To test the procedure in such a circumstance, we introduce biased errors into the Langevin-simulated system described above in Fig. S1. The biased errors are modeled as skewed normal distributions with constant, positive skewness of 0.67 and a normal error magnitude (i.e., standard deviation) of 0.1. The bias (skewness) remains unknown in the analysis, as the error distributions are assumed to be normally distributed (with zero skewness) with an error magnitude of 0.1. The assumed (blue) and true (black) error distributions are shown alongside one another in Fig. S6A. The effects of biased error on the true state distributions are shown in Fig. S6B, where the biased error state distributions (solid) differ from the unbiased state distributions (dashed).

As shown in Fig. S6C, in which the true state distributions (dashed) are shown alongside the SCISM-extracted state distributions (solid), the SCISM procedure extracts the appropriate number of states and accurately extracts their distributions, all states having overlap coefficients greater than 0.95 with a segment length of 100 ms. Figs. S6D-S6F show true state assignments along the time series, the SCISM-assigned maximum conditional probability assignments, and the magnitudes of the conditional probabilities, respectively. As shown by the trajectories, the SCISM assigned states are largely accurate,

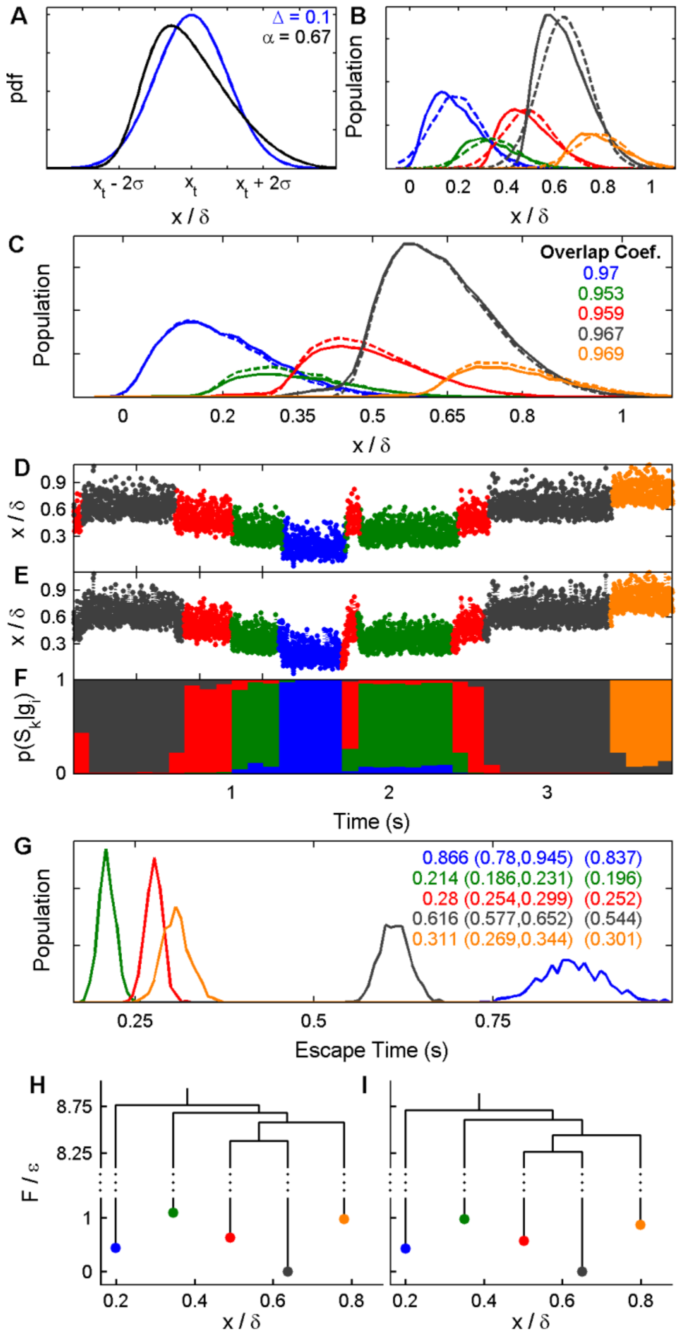

Figure S6. SCISM and Biased Error. **A)** The true (black) and assumed (blue) error distributions. The error magnitude Δ and the skewness α are also shown. **B)** Biased error (solid) and unbiased error (dashed) state distributions. **C)** True (dashed) and extracted (solid) state distributions. Representative trajectories with **D)** true state assignments and **E)** maximum probability state assgnments. **F)** Bar heights denote the magitudes of the conditional probabilities. **G)** Extracted escape time distributions. **H)** Extracted TRDG. **I)** True TRDG.

except in segments that contain short residencies and transitions. Even in such segments however, the conditional probabilities in Fig. S6F reflect the correct state assignments through their magnitudes. For example, although the maximum probability assignment of the first segment misses the presence of the state shown in red, the conditional probability of this state at this segment is significant, meaning the short residence time will be represented in the escape time calculation. This observation is confirmed in the escape time distributions shown in Fig. S6G, as the true escape times are largely within the confidence limits of the extracted escape times. Furthermore, as shown by Figs. S6H and S6I containing the extracted and true TRDGs, respectively, the effective free energy landscape is also faithfully recovered.

Though bias in the measurement error is not always constant as it is in this model, the results presented in Fig. S6 offer encouraging evidence towards the resistance of the SCISM procedure to unknown error contributions. Indeed, when presented with an unknown, systematic error contribution, SCISM performs more than adequately, accurately extracting many of the system's underlying properties.

Section S3: Results for a Simulated SM FRET System

To validate the SCISM procedure in its application to a SM FRET system, test trajectories emulating a SM FRET experiment were constructed via photon-by-photon Monte Carlo simulation.[1] The system contained 3 states whose mean FRET efficiencies E_i were 0.25, 0.5, and 0.75, respectively. The states were arranged in a linear transition network, i.e., there were no transitions between the states having efficiencies 0.25 and 0.75. Input transition rate constants were 2 Hz from the states 0.25 and 0.75, and 1 Hz from the central state at 0.5 efficiency. The escape rate k_i from a state i was taken to be the sum of the outgoing rate constants, i.e., $k_i = \sum_{j \neq i} k_{ij}$. Here $k_{ij} = P(\text{state } j|\text{state } i)$ is the transition probability per unit time from state i to j. Dwell times in each state were generated with Monte Carlo methods, e.g., a particular dwell time τ_{dwell} was generated with the following Eq. S4, with rand representing a random number sampled from the uniform distribution on the interval (0,1).

$$\tau_{\text{dwell}} = -\log(\text{rand})/k_i \qquad \text{(S4)}$$

Note that Eq. S4 generates Markovian escape kinetics whose dwell time distributions are single exponential. Each trajectory was generated independently, with the initial state being selected at random. The length of each trajectory is determined by photo-bleaching of dye molecules whose kinetics also conform to a single exponential decay function with a mean length τ_{bleach} of 3 s. The length of each trajectory, t_{bleach}, was randomly selected as well.

$$t_{\text{bleach}} = -\tau_{\text{bleach}} \log(\text{rand}) \qquad \text{(S5)}$$

To accurately mimic typical single FRET measurements, the total number of donor and acceptor photons per 2 ms time bin was input to average 40 photons. This results in average total intensity $I_{\text{tot}} = I_A + I_D$ = 20,000 photons/s, where I_A is the average acceptor intensity and I_D the average donor intensity. This average total intensity I_{tot} was varied for each trajectory by sampling from a normal distribution with mean I_{tot} and standard deviation $0.1 \cdot I_{\text{tot}}$. Intensities of donor and acceptor photons were calculated using the mean efficiency E_i of the state i, i.e., $I_A^{(i)} = E_i \cdot I_{\text{tot}}$ and $I_D^{(i)} = I_{\text{tot}}(1 - E_i)$. Arrival times for each

photon were generated stochastically using Poisson statistics, i.e., the arrival time t_k for the kth emitted acceptor photon was generated with the following set of equations:

$$\Delta t = -\log(\text{rand})/I_{\text{A}}{}^{(i)}, \tag{S6a}$$

$$t_k = t_{k-1} + \Delta t. \tag{S6b}$$

The initial time t_0 was set to zero at the initiation of each trajectory. To simulate the donor-to-acceptor crosstalk encountered in FRET measurements, a 10% donor to acceptor crosstalk probability was included, i.e., each emitted donor photon has a 10% probability to be detected on the acceptor channel. The arrival times of these donor-emitted photons were recorded as acceptor photons. Because the probabilities of acceptor-to-donor crosstalk are typically small (< 1%),[2] acceptor-to-donor crosstalk was not included in this simulation. Background photons were also included for both the acceptor and donor channels, $I_{\text{A}}{}^{(\text{b})}$ and $I_{\text{D}}{}^{(\text{b})}$, respectively, with a signal-to-background ratio of ~5, e.g., $I_{\text{A}}{}^{(\text{b})} = I_{\text{D}}{}^{(\text{b})} = 0.1 \cdot I_{\text{tot}}$. Arrival times for background photons were generated using Eqs S6.

SM FRET experiments return time series of observed acceptor and donor photon counts that contain flurorphore emission, background, and crosstalk photons. To simulate these measurements, we generate 'observed' acceptor and donor photon counts, N_{A} and N_{D}, respectively. After the arrival times were generated, the photons were uniformly binned to 2 ms to generate the 'observed' N_{A} and N_{D}. The numbers of photons at a particular bin were calculated with the following equations:

$$N_{\text{A}} = N_{\text{A}}{}^{(\text{em})} + B_{\text{A}}, \tag{S7a}$$

$$N_{\text{D}} = N_{\text{D}}{}^{(\text{em})} + B_{\text{D}}. \tag{S7b}$$

Here, $N_{\text{A}}{}^{(\text{em})}$ includes the acceptor-emitted photons as well as the donor-emitted crosstalk photons, B_{A} is the number of background photons detected on the acceptor channel in the time bin, $N_{\text{D}}{}^{(\text{em})}$ is the number of donor-emitted photons, not including the crosstalk photons, and B_{D} is the number of background photons detected on the donor channel during the course of the time bin. In order to calculate a FRET efficiency at each time bin, the 'observed' photon counts, N_{A} and N_{D}, must be corrected for background and crosstalk. For this, we averaged the numbers of acceptor (B_{A}) and donor (B_{D}) background photons per time bin over the simulated trajectory, returning $\langle B_{\text{A}} \rangle$ and $\langle B_{\text{D}} \rangle$, respectively. The estimated number of crosstalk photons per time bin is $N_{\text{X}} = \chi(N_{\text{D}} - \langle B_{\text{D}} \rangle)$ where $\chi = x/(1-x)$ with x being the crosstalk probability, 0.10. (The crosstalk probability can be evaluated in a separate SM experiment using only donor fluorophores). Using these quantities, we estimate the apparent numbers of acceptor-emitted and donor-emitted photons per time bin, n_{A} and n_{D}.

$$n_{\text{A}} = N_{\text{A}} - \langle B_{\text{A}} \rangle - N_{\text{X}} \tag{S8a}$$

$$n_{\text{D}} = N_{\text{D}} - \langle B_{\text{D}} \rangle + N_{\text{X}} \tag{S8b}$$

After the apparent fluorophore-emitted photon counts are obtained, the apparent efficiency at the time bin, E_{A} is calculated with the standard FRET efficiency equation.

$$E_{\text{A}} = \frac{n_{\text{A}}}{n_{\text{A}} + n_{\text{D}}} \tag{S9}$$

A total of 50 trajectories were simulated in this manner, resulting in 68,624 data points in 2 ms time bins. The trajectories resulted in 1,004 segments after SCISM implementation with a segment length of 100

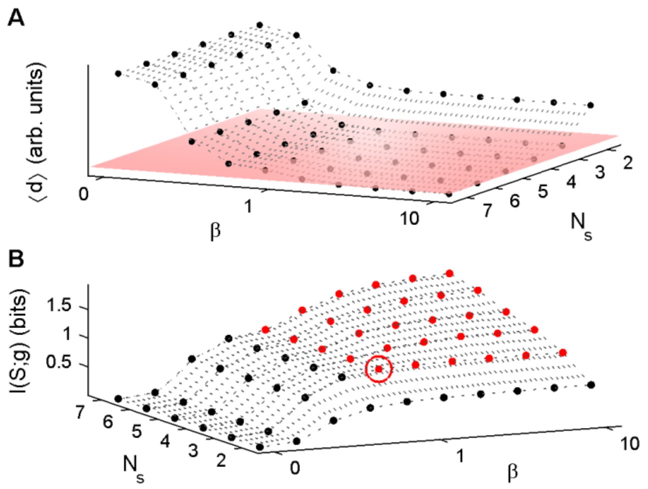

Figure S7. A) Mean distortion vs. the number of states N_s and the value of the Lagrange multiplier β. The horizontal surface represents the amount of distortion due to error. B) The information $I(\boldsymbol{S};\boldsymbol{g})$ vs. β and N_s. Models falling below the distortion due to error are highlighted in red. The selected model, having the smallest $I(\boldsymbol{S};\boldsymbol{g})$ while satisfying the distortion requirement, is indicated with a red circle.

ms. The segments were taken sequentially to avoid overlap between segments, and segments did not span multiple trajectories. Extraneous time bins at the end of a trajectory were not included in the analysis.

Minimizing Eq. 1 of the main text for each value of β and N_S results in a soft clustering that returns $p(S_k|g_i)$ for all segments and states and, hence, returns the mean distortion $\langle d \rangle$. The results returned for the set of β and N_S give rise to a set of models describing the system, from which we must select the most appropriate model. To visualize the behavior of the mean distortion $\langle d \rangle$ and determine the appropriate values of β and N_S, we plot the $\langle d \rangle$ as a function of β and N_S in the upper panel of Fig. S7A (The same procedure is carried out for single-molecule time series of the AMPA ABDs while bound to glutamate, nitrowillardiine, and UBP282). The mean distortion $\langle d \rangle$ is calculated from the Kantorovich distances d_{ij} between all segments g_i and g_j. The segments contributing to $\langle d \rangle$ are constructed from finite numbers of data points that contain experimental error, thus we must account for sampling error as well as the experimental error in calculating $\langle d \rangle$. Here we calculate many bootstrapped segments $g_i{}'$ and $g_j{}'$ having the same number of data points as the original segments, which corresponds to performing many "observations". From these bootstrapped $g_i{}'$ and $g_j{}'$, we elucidate the 95% error intervals in the distribution of $\langle d \rangle$ at each β and N_S. These errors are contained within the size of the markers for each model in Fig. S7. (See also *Convergence of the Bootstrap Distributions* below). Looking at, for instance, $\beta > 0.5$, we observe that $\langle d \rangle$ decreases as N_s increases, indicating that as the number of states increases, the mean distortion within each state averaged over all the states decreases. In contrast, for $\beta \ll 0.5$ the minimization with respect to mean distortion becomes irrelevant to minimizing Eq. 1, as the value of β is too small for the distortion term to contribute substantially to the functional. In Fig. S7A the horizontal and partially transparent red plane indicates the distortion cutoff described in Methods in the

main text. This cutoff represents the distortion below which experimental and sampling errors dominate the observed distortion. The plane intersects the distortion surface $\langle d\rangle(\beta, N_s)$, indicating that the distortion arising in the models lying below the plane is explained by error. These models are highlighted in red markers in the information surface $I(\boldsymbol{S};\boldsymbol{g})$ of Fig. S7B. The values of $I(\boldsymbol{S};\boldsymbol{g})$ arising from each of the models reveal that the simplest model having distortion below the error cutoff has $N_s = 3$ and $\beta \approx 1$, as indicated by the red circle. That is, among the red markers, $N_s = 3$ and $\beta \approx 1$ is the desired minimal model because the compression, quantified by the magnitude value of the mutual information $I(\boldsymbol{S};\boldsymbol{g})$, is maximized (i.e., $I(\boldsymbol{S};\boldsymbol{g})$ is minimized). This model is then the appropriate model for further analysis, as shown in Fig. S8.

Fig. S8A plots the simulated distribution of each underlying state (solid lines) along with the distributions extracted via SCISM (dashed lines). The agreement between the true efficiency distribution

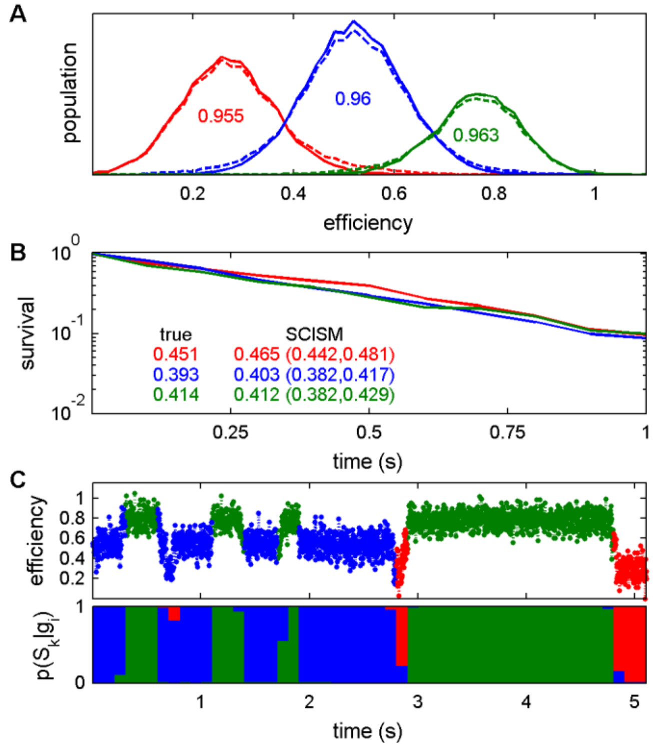

Figure S8. A) True (solid) and SCISM-extracted (dashed) state distributions. Numbers are the values of the overlap integral between the true and extracted distributions. B) True (solid) and SCISM-extracted (dashed) survival curves with the y-axis on a log scale. Escape times are reported in seconds. The numbers in the parenthesis are the lower and upper bound of 95% error interval. Colors correspond to those in A. C) (upper panel) A representative trajectory from the three-state simulation, colored by most probable state (via $p(S_k|g_i)$). Colors correspond to those in B and C. (lower panel) Bar heights correspond to the conditional probabilities of each cluster given each segment.

and the SCISM-extracted distribution of the ith state is quantified with the overlap coefficient O_i as described in Eq. S3, where the summation is taken over the efficiency coordinate. Values of the O_i, shown in corresponding color inside each state distribution, confirm the agreement between the extracted and true distributions, yielding a maximum deviation from unity of 4.5%. Fig. S8B displays the survival curves (see below for a description of survival curve and escape time estimation) resulting from SCISM analysis of the simulated system on a logarithmic y-axis to display their linearity. The escape times of each state, displayed in seconds and in corresponding color, are in good agreement with their true counterparts, as the true values lie well within the 95% error intervals of each extracted lifetime. The upper panel of Fig. S8C shows a representative trajectory from the simulated data set. Each segment's color corresponds to the most probable state at that segment. Colors correspond to those of the state distributions in Fig. S8A. Bar heights in the lower panel of Fig. S8C show the conditional probability of each state at each segment. Here we note the utility of soft clustering by observing that transitions occur within some segments. Assignment of these segments to only one state is inappropriate considering that they contain contributions from two or more underlying states. Soft clustering allows the segments containing transitions to be assigned to multiple underlying states, thereby improving the quality of our description. Overall, Figs. S7 and S8 demonstrate that, given a simulated, three-state SM FRET system, SCISM appropriately and, equally importantly, automatically identifies and characterizes the states underlying the simulated data.

Section S4: Survival Curves and Escape Times for AMPA ABDs

Survival curves and escape times were estimated from state sequences, e.g., the assignment of a particular state at each segment in each trajectory. As discussed in the main text, many state sequence realizations can be constructed owing to our use of soft clustering. Specifically, a state is assigned to a particular segment by generating a uniformly distributed random number and comparing that number to the conditional probabilities $p(S_k|g_i)$ of each state. Because the $p(S_k|g_i)$ are normalized over S_k for each g_i, the first state having cumulative probability $\sum_{k' \leq k} p(S_{k'}|g_i)$ greater than the random number is selected as the state for the segment g_i. For example, given a particular segment and the set of states $\{S_1, S_2, S_3\}$ with conditional probabilities $\{0.1,\ 0.6,\ 0.3\}$, we have cumulative probabilities $\{0.1,\ 0.7,\ 1\}$. We generate a uniformly distributed random number on (0,1), say 0.57. We then compare 0.57 to the cumulative probabilities and assign the state S_2 to this particular segment. Repeating this process a number of times for all segments in all trajectories generates a collection of state sequence realizations that characterize the uncertainties in state assignments. Properties that are subsequently estimated from a state sequence, such as survival curves and escape times, are thus estimated from each realization, yielding errors associated with each property.

Dwell times in each state are compiled in order to estimate survival curves and escape times. (Here 'escape time' refers to the average amount of time the system resides in a particular state prior to transition to any of the other states. 'Dwell time' refers to a single instance of residence in a particular state prior to a transition.) After the dwell times have been compiled, the maximum likelihood estimates of the escape times correspond simply to the means of each of the states' dwell times.[3] Survival curves $S(\tau)$ are estimated by constructing the cumulative distribution of dwell times $C(\tau)$ versus time, then taking the difference $S(\tau) = 1 - C(\tau)$. These actions are performed for each of 1,000 state sequence

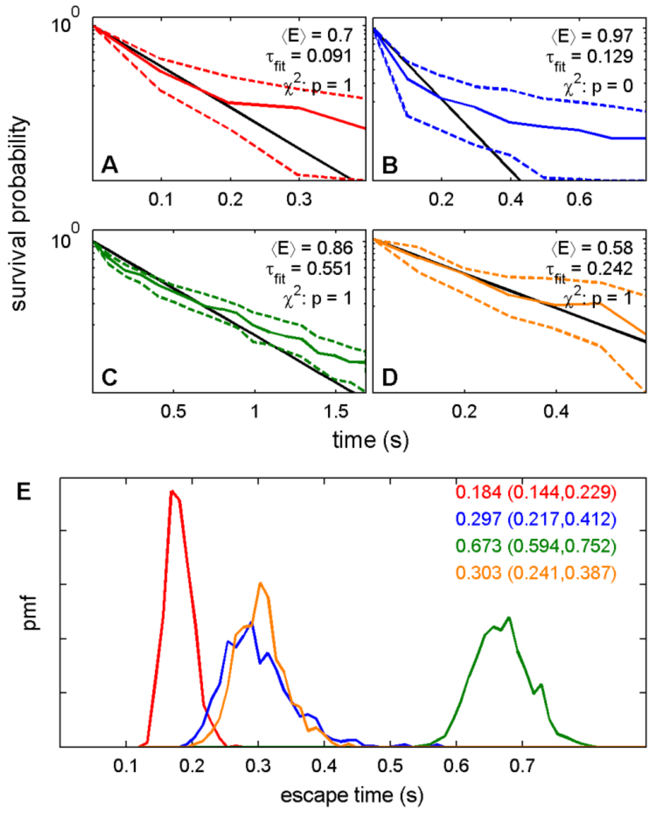

Figure S9. Survival curves and escape time distributions for the glutamate-bound ABDs. A)-D) Most probable survival curves (solid lines) are displayed along with the 95% confidence intervals (dashed lines) and the single-exponential fitted curve (solid black lines). Mean efficiencies, fitted decay times, and *p*-values are also included. E) Escape time distributions for each of the extracted states. Most probable escape times and the 95% confidence intervals are displayed in corresponding colors. Note that all colors correspond to those in Fig. 2 of the main text.

realizations, yielding 1,000 survival curves and escape times for each state. Errors for the escape times are reported as the 95% confidence interval of each escape time distribution, and errors on the survival curves are displayed as the 95% confidence interval at each time step. Time steps for survival curves are 99 ms, equivalent to the segment lengths. Single exponential behavior is tested via the survival curves. The most probable escape times from the escape time distributions are used to generate the single-exponential survival curves shown in black in Figs S9-S11. A χ^2-test for goodness-of-fit is then performed, yielding a *p*-value for each of the most probable survival curves. It is of note that all χ^2-tests reported here returned *p*-values of either 0 or 1. Figs. S9-S11 display survival curves and escape times for each extracted state in each of the glutamate-bound, nitrowillardiine-bound, and antagonist-bound AMPA ABDs, respectively.

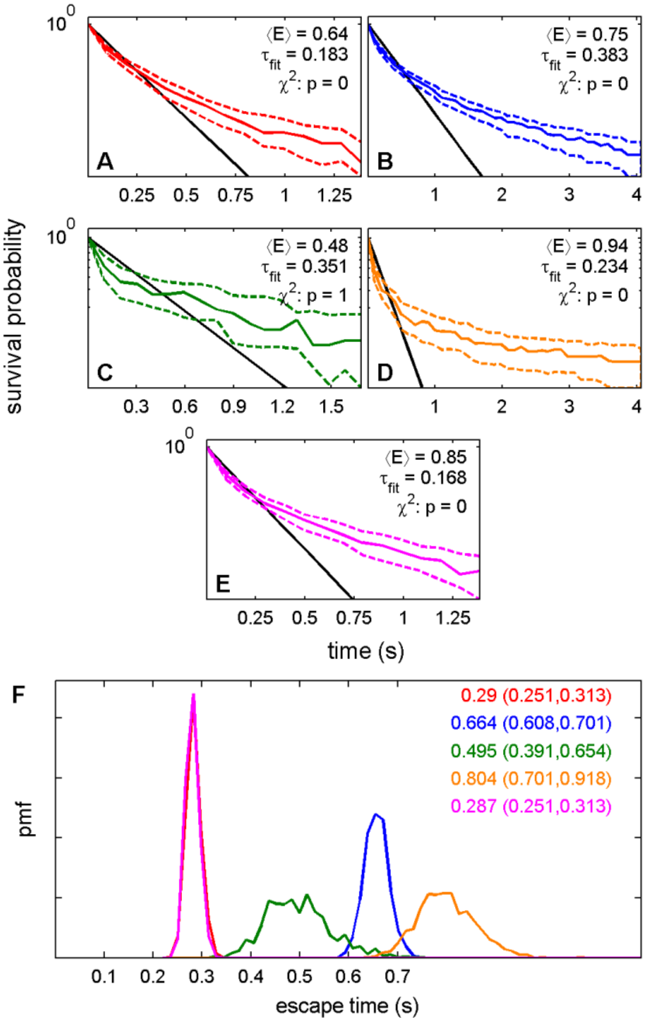

Figure S10. Survival curves and escape time distributions for the nitrowillardiine-bound ABDs. See the caption of Fig. S9 for a description of the contents.

Section S5: Testing the Steady-State Networks for Detailed Balance

We test detailed balance for each pair of states in our systems using a simple binomial test. Taking the null hypothesis to be the existence of detailed balance among all states, we test it against the alternative hypothesis that $N_{ij} = k$ observed transitions violates the detailed balance hypothesis for a given number of transitions $N_{\text{tot}} = N_{ij} + N_{ji}$. The binomial probability of k transitions in N_{tot} trials with equal forward and backward transition probabilities is

$$P(k) = \binom{N_{\text{tot}}}{k} p^k (1-p)^{N_{\text{tot}}-k} = \binom{N_{\text{tot}}}{k} \left(\frac{1}{2}\right)^k \left(\frac{1}{2}\right)^{N_{\text{tot}}-k} = \binom{N_{\text{tot}}}{k} \left(\frac{1}{2}\right)^{N_{\text{tot}}}. \tag{S10}$$

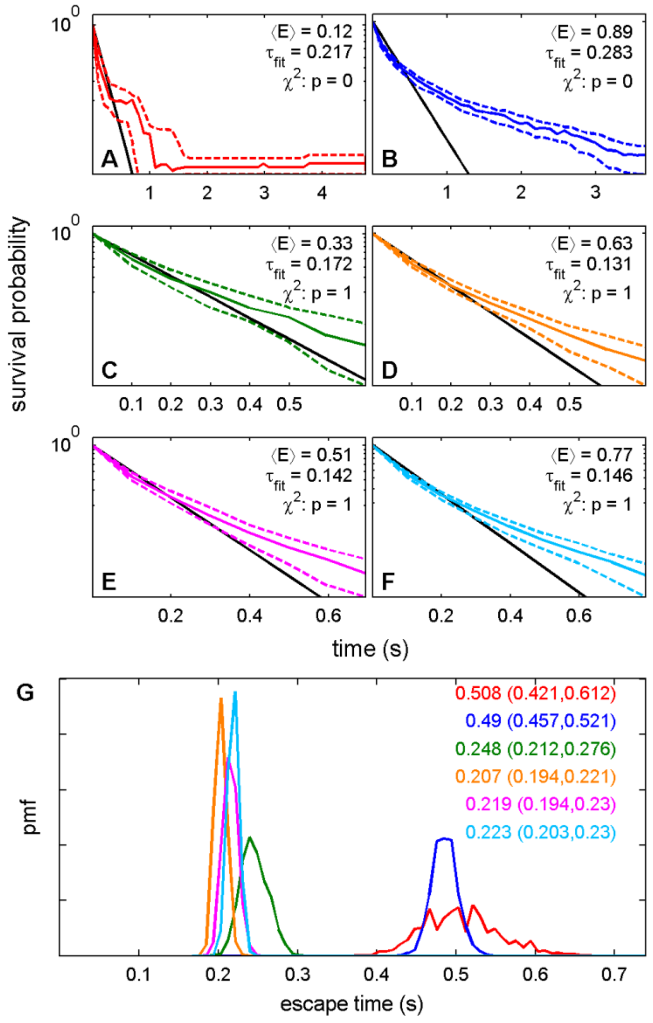

Figure S11. Survival curves and escape time distributions for the UBP282 antagonist-bound ABDs. See the caption of Fig. S9 for a description of the contents.

If the cumulative probability $\mathrm{P}(k' \leq k) = \sum_{k'=0}^{k} P(k')$ is greater than a significance level α (e.g., 5%), then the null hypothesis cannot be rejected. Conversely, if $\mathrm{P}(k' \leq k) \leq \alpha$ then the null hypothesis is rejected and detailed balance is violated.

Detailed balance hypothesis tests for the transition networks returned for the simulated system, as well as the glutamate-bound, nitrowillardiine-bound, and UBP282-bound systems were performed. It was found that none of these systems violates the detailed balance hypothesis, thus our steady-state networks are regarded as equilibrium networks. Because our steady-state networks uphold detailed balance at the time scale of τ_{seg}, the protocol of Krivov and Karplus[4,5] was applied to the equilibrium networks.

It should, however, be noted that even if detailed balance does not hold for each pair of states in a network, the stationary state condition guarantees that detailed balance holds between any two disjoint subsets of nodes as proved by Gupta.[6] Therefore, TRDG developed by Krivov and Karplus[4,5] can be applied not only to equilibrium networks but also any steady-state network, which takes into account all

possible pathways in the network and is free from any artifacts of the projection into one-dimensional coordinate. However, if the system is not in equilibrium but in the steady-state condition in which detailed balance does not hold for all pair of nodes in the network, one should discuss not only the free energy landscape but also flux over the landscape.[7,8]

Section S6: Convergence of the Bootstrap Distributions

As discussed in Section S3, in order to deal with sampling error arising from the finiteness of data points, many bootstrapped short time distributions g_i' and g_j' are generated. However, how many bootstrapped short time distributions should be generated? To ensure the convergence of the bootstrapped mean distortion $\langle d \rangle$ distributions, as well as to fix the number of bootstrapped samples for each clustering result, we use a series of two-tailed, two-sample Kolmogorov-Smirnov (K-S) hypothesis tests. We begin by bootstrapping all segments 25 times, and use the bootstrapped segments to calculate 25 bootstrapped mean distortions via Eq. 3 in the main text. We then generate 25 additional bootstrapped samples and append those to the original 25. A K-S test is then performed with the null hypothesis being that the populations containing 25 and 50 bootstraps are sampled from the same underlying distribution at a type I error rate of 5%. If the null hypothesis is accepted, the procedure is terminated and the better statistics of the 50-sample population are retained. If the null hypothesis is rejected, 25 additional bootstraps are generated and appended to the 50-sample population, and another K-S test is performed. This procedure continues until the null hypothesis is accepted, at which point the population containing the larger number of samples is retained. The procedure is illustrated in the accompanying Fig. S12, in which the data from the three-state simulation described in Section S3 was used as an example. It is clearly shown that, although 25 bootstraps are insufficient for convergence of the distribution, numbers of bootstraps that are ≥ 50 are sufficiently converged in comparison to a population containing 1,000 bootstrapped distortions.

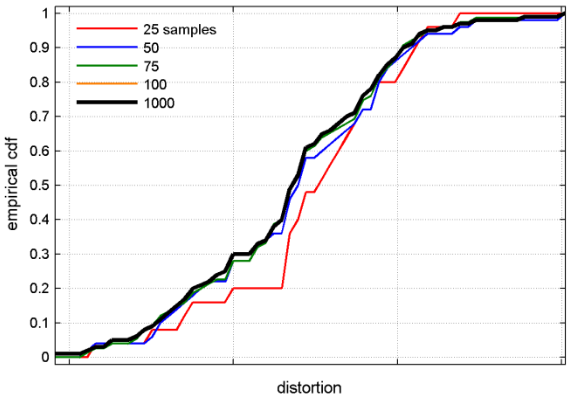

Figure S12. Convergence of bootstrapped distortion distributions as the number of bootstrapped samples increases.

Section S7: Self-Distortion Due to Error

As discussed in the main text, distortion arises within a particular segment due to empirical and sampling errors. Furthermore, we may turn this phenomenon to our advantage and use it to select the appropriate model describing the system, thus maximizing the information contained within the data. We illustrate this self-distortion in Fig. S13. Fig. S13A shows a typical trajectory from the glutamate-bound ABD data set. (Note this trajectory is also shown in Fig. 3A of the main text.) Error bars correspond to empirical errors. To illustrate intra-segment distortion arising from errors, we focus on the segment highlighted in blue in Fig. S13A. Using the bootstrapping procedure described above, we construct two realizations of the segment pmf as shown in Fig. S13B. Finally, Fig. S13C shows the cumulative distribution functions (cdfs) of the two probability mass functions (pmfs) shown in Fig. S13B. As illustrated by the shaded region, the Kantorovich distance between the two realizations of the highlighted segment is nonzero, thus illustrating the intra-segment distortion arising from errors.

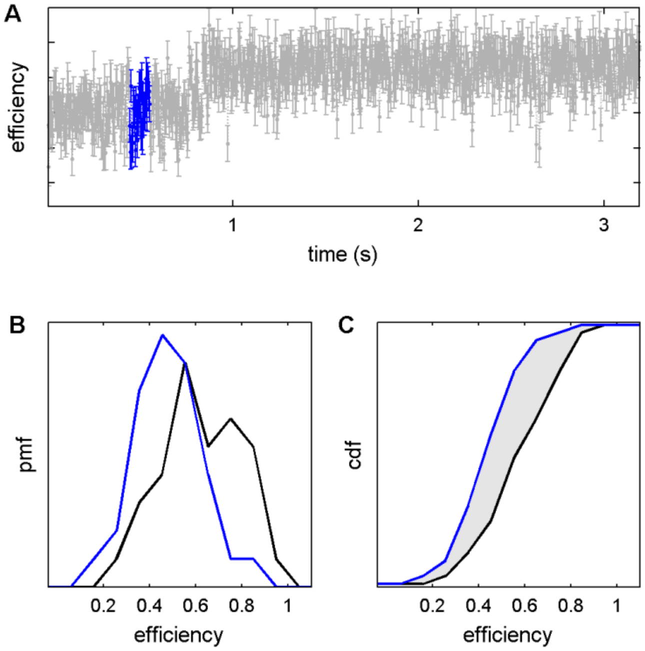

Figure S13. Self-distortion due to error. A) A typical trajectory from the glutamate-bound ABD data set. B) Two bootstrapped pmfs arising from the segment highlighted in blue. C) The cdfs of the tow pmfs shown in B, with the shaded region illustrating nonzero distortion between the two cdfs.

Section S8: The Kantorovich Distance and Degeneracy Lifting

Degenerate states, e.g., two states having the same mean or falling in the same range along the observable coordinate, pose potential issues in their differentiation for traditional state identification methods such as maximum likelihood hidden Markov models.[9] In this section we demonstrate the capability of the Kantorovich distance used along with the segmentation procedure in differentiating degenerate states. Consider the toy trajectory shown in Fig. S14A. This trajectory was constructed to have contributions two distinct states, denoted by the black and blue regions of the trajectory, having the

same mean but different variances along the observable coordinate. The pmfs for each state are shown in Fig. S14B, and the corresponding cdfs are shown in Fig. S14C. As is shown in Fig. S14C, the Kantorovich distance, i.e., the area of the shaded region between the two curves, between these two states is nonzero, thereby allowing for their differentiation in the clustering procedure.

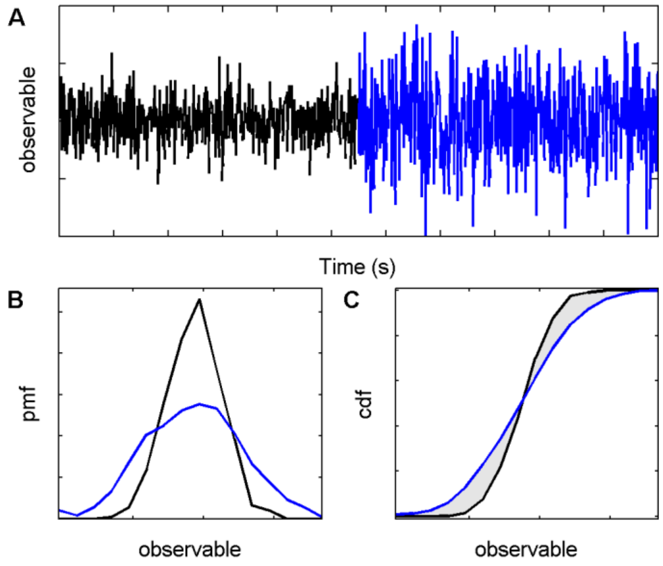

Figure S14. Degeneracy lifting and the Kantorovich distance. **A)** A toy trajectory containing two distinct states (black, blue) having the same mean but different variance along the observable coordinate. **B)** The pmfs of the black and blue regions of the trajectory. **C)** The cdfs of the black and blue regions of the trajectory. The area of the shaded region between the cdfs is the nonzero Kantorovich distance.

References

1. Taylor, J. N. & Landes, C. F. Improved Resolution of Complex Single-Molecule FRET Systems via Wavelet Shrinkage. *J. Phys. Chem. B* **115,** 1105–1114 (2011).

2. Landes, C. F., Rambhadran, A., Taylor, J. N., Salatan, F., & Jayaraman, V. Structural landscape of isolated agonist-binding domains from single AMPA receptors. *Nat Chem Biol* **7,** 168–173 (2011).

3. Terentyeva, T. G. *et al.* Dynamic Disorder in Single-Enzyme Experiments: Facts and Artifacts. *ACS Nano* **6,** 346–354 (2011).

4. Krivov, S. V. & Karplus, M. Free energy disconnectivity graphs: Application to peptide models. *J. Chem. Phys.* **117,** 10894–10903 (2002).

5. Krivov, S. V. & Karplus, M. Hidden complexity of free energy surfaces for peptide (protein) folding. *Proc. Natl. Acad. Sci. U. S. A.* **101,** 14766–14770 (2004).

6. Gupta, R.P. On Flows in Pseudosymmetric Networks. *SIAM Journal on Applied Mathematics*, **14**, 215-225 (1966).

7. Xu L, Zhang F, Wang E, & Wang J. The potential and flux landscape, Lyapunov function and non-equilibrium thermodynamics for dynamic systems and networks with an application to signal-induced Ca^{2+} oscillation. *Nonlinearity*, **26**, R69–R84 (2013).

8. Wang, J., Li, C.H., & Wang, E.K. Potential and flux landscapes quantify the stability and robustness of budding yeast cell cycle network. *Proc. Natl. Acad. Sci. U.S.A* **107**, 8195–8200 (2010).

9. McKinney, S. A., Joo, C., & Ha, T. Analysis of Single-Molecule FRET Trajectories Using Hidden Markov Modeling. *Biophys. J.* **91,** 1941–1951 (2006).
